# The combination of plant-expressed cellobiohydrolase and low dosages of cellulases for the hydrolysis of sugar cane bagasse

**DOI:** 10.1186/s13068-014-0131-9

**Published:** 2014-09-09

**Authors:** Mark D Harrison, Zhanying Zhang, Kylie Shand, Barrie Fong Chong, Jason Nichols, Paul Oeller, Ian M O’Hara, William OS Doherty, James L Dale

**Affiliations:** Syngenta Centre for Sugarcane Biofuels Development, Queensland University of Technology, GPO Box 2432, 2 George Street, Brisbane, Queensland 4001 Australia; Centre for Tropical Crops and Biocommodities, Queensland University of Technology, GPO Box 2432, 2 George Street, Brisbane, Queensland 4001 Australia; Syngenta Biotechnology Inc., Research Triangle Park, 3054 East Cornwallis Road, Durham, NC 27709-2257 USA

**Keywords:** Cellulase, Cellobiohydrolase, Transgenic, Sugar cane, Biomass, Pretreatment, Enzymatic hydrolysis, Saccharification

## Abstract

**Background:**

The expression of biomass-degrading enzymes (such as cellobiohydrolases) in transgenic plants has the potential to reduce the costs of biomass saccharification by providing a source of enzymes to supplement commercial cellulase mixtures. Cellobiohydrolases are the main enzymes in commercial cellulase mixtures. In the present study, a cellobiohydrolase was expressed in transgenic corn stover leaf and assessed as an additive for two commercial cellulase mixtures for the saccharification of pretreated sugar cane bagasse obtained by different processes.

**Results:**

Recombinant cellobiohydrolase in the senescent leaves of transgenic corn was extracted using a simple buffer with no concentration step. The extract significantly enhanced the performance of Celluclast 1.5 L (a commercial cellulase mixture) by up to fourfold on sugar cane bagasse pretreated at the pilot scale using a dilute sulfuric acid steam explosion process compared to the commercial cellulase mixture on its own. Also, the extracts were able to enhance the performance of Cellic CTec2 (a commercial cellulase mixture) up to fourfold on a range of residues from sugar cane bagasse pretreated at the laboratory (using acidified ethylene carbonate/ethylene glycol, 1-butyl-3-methylimidazolium chloride, and ball-milling) and pilot (dilute sodium hydroxide and glycerol/hydrochloric acid steam explosion) scales. We have demonstrated using tap water as a solvent (under conditions that mimic an industrial process) extraction of about 90% recombinant cellobiohydrolase from senescent, transgenic corn stover leaf that had minimal tissue disruption.

**Conclusions:**

The accumulation of recombinant cellobiohydrolase in senescent, transgenic corn stover leaf is a viable strategy to reduce the saccharification cost associated with the production of fermentable sugars from pretreated biomass. We envisage an industrial-scale process in which transgenic plants provide both fibre and biomass-degrading enzymes for pretreatment and enzymatic hydrolysis, respectively.

**Electronic supplementary material:**

The online version of this article (doi:10.1186/s13068-014-0131-9) contains supplementary material, which is available to authorized users.

## Background

Plant biomass is the primary source of simple sugars, and bacterial, fungal, and algal fermentation enable the transformation of these sugars into a wide range of renewable platform chemicals, fuels, and value-added products. Most of these products, including fuel ethanol, are currently produced by the fermentation of simple sugars naturally present in sugar cane juice or molasses, or derived from the enzymatic hydrolysis of corn starch [1]. Lignocellulosic biomass such as sugar cane fibre (bagasse) and corn stover, forestry and municipal wastes, and dedicated energy crops are primarily composed of plant cell walls and can also serve as a renewable source of fermentable sugars. These second generation feedstocks constitute a global carbohydrate resource and are typically of lower cost than first generation feedstocks, starch and sugar [2]. However, accessing these sugars is not simple, because the plant cell wall is a resilient, intractable barrier composed of complex polysaccharides (cellulose and hemicellulose), lignin, and proteins. Cellulose is an unbranched, homopolysaccharide polymer consisting of D-anhydroglucose repeating units joined by 1,4-β-D-glycosidic linkages. Cellulose in the plant cell wall is ordered into fibrils within a matrix containing lignin and hemicellulose. Most cellulose in plant biomass is crystalline, while the remainder is amorphous [3]. Hemicellulose is a heterogeneous, branched heteropolysaccharide consisting of C5 sugars (xylose, arabinose), C6 sugars (mannose, glucose, and galactose), and uronic acids. Hemicellulose provides the connection between lignin and the cellulose fibres, and gives the polymer network rigidity [4]. Lignin is a complex molecule containing cross-linked polymers of phenolic monomers with both aliphatic and aromatic constituents [5]. Lignin is totally amorphous and hydrophobic [6]. Cellulose is embedded in the matrix containing hemicellulose and lignin, and pretreatment is essential in order to make it more accessible for efficient enzymatic hydrolysis to glucose [7]. Ongoing technology improvements have substantially reduced the cost of biomass pretreatment and enzymatic hydrolysis to fermentable sugars for bioethanol production; however, recent estimates still indicate that these processes account for about 30% of the total process cost [8]. The cost of enzymatic hydrolysis mainly depends on the enzyme dosage and the hydrolysis rate, which in turn depends on cellulose accessibility to enzymes.

Cellulose is enzymatically hydrolysed by highly specific enzymes that are produced naturally by a wide range of bacteria and fungi [9], although relatively few microbes secrete enzyme mixtures capable of complete cellulose saccharification [10]. Fungus-derived commercial cellulase mixtures contain numerous enzymes and non-catalytic proteins that together hydrolyse not only cellulose, but also hemicellulose [11]. A minimum of four cellulases is required for complete cellulose saccharification, and each is derived from a different functional class: (i) an endo-1,4-β-D-glucanase (endoglucanase) that hydrolyses cellulose regions with low crystallinity (called amorphous regions), creating free chain ends; (ii) two exo-1,4-β-D-glucanases (cellobiohydrolases (CBHs)), that cleave cellobiose units from either the reducing or non-reducing free chain ends; and (iii) a β-glucosidase (βG) that hydrolyses cellobiose to glucose [12]. CBHs catalyse the majority of bond cleavages during cellulose hydrolysis and are usually the major component of fungus-derived commercial cellulase mixtures [13] or artificial enzyme mixtures for cellulose hydrolysis [14–20].

There are numerous studies describing the hydrolysis of pretreated biomass by mixtures of purified cellulases, including barley straw [16], wheat straw [18,19], corn stover [15,17,20], switchgrass [15,21], *Miscanthus* [15], willow [22], poplar [15,21], and Douglas fir wood [14]. These studies have clearly demonstrated that the choice of pretreatment process has a profound effect on the overall susceptibility of a given biomass to hydrolysis by commercial cellulase mixtures or mixtures of purified cellulases. In fact, we recently reported that glucan conversions obtained with mxtures of *Trichoderma reesei* CBH I and βG on bagasse pretreated at the pilot scale with acidified glycerol were 10 to 30% higher than those obtained with NaOH or H_2_SO_4_ pretreatments [23]. However, the use of plant-expressed CBH as an additive to a commercial cellulase mixture may offer the best option to substantially reduce the costs of enzymes for lignocellulosic saccharification [24–26]. There are few studies describing the ability of plant-expressed cellulase to hydrolyse cellulose in pretreated biomass, either alone [27–30] or in combination with sub-optimal doses of commercial cellulase mixtures [31]. The present study complements these studies and is an extension of our previous work [23], wherein we examined saccharification of bagasse pretreated using dilute NaOH, H_2_SO_4_, HCl, and acidified glycerol at the pilot scale. Herein, we have assessed plant-expressed CBH as an additive to a commercial cellulase for saccharification of these pretreated bagasse substrates and bagasse pretreated using a novel, acid-catalysed ethylene carbonate (EC)/ethylene glycol (EG) mixture, 1-butyl-3-methylimidazolium chloride (BMIMCl), and ball-milling. This approach provided substrates with a range of compositional and structural features in order to assess the selectivity of plant-expressed CBH. Furthermore, this study investigated the extraction of the plant-expressed CBH using a simple mechanical treatment process, with water as the solvent, that is suitable in an industrial-scale process.

## Results

### Expression of recombinant CBH transgenic corn

The CBH utilised in this study (Additional file 1: Figure S1) is a proprietary biomolecule obtained from Verenium Corporation [32] that shares 96% amino acid sequence identity with CBH I from *Penicillium occitanis* [33] and 61% amino acid sequence identity with CBH I (Cel7A) from *Trichoderma reesei* [34]. The expression of CBH in corn in the present study was under the control of the green-tissue-specific phosphoenolpyruvate carboxylase (*Zm*-*PepC*) promoter [35]. Embryogenic callus from corn (*Zea mays* inbred variety AX5707) was transformed by *Agrobacterium*-mediated transformation, and at least 40 independent transgenic events were regenerated. Initial transgenic (T_0_) events were screened for cellulase accumulation, and 30 elite events were crossed with maize variety ID5829 to produce F1 seed for subsequent experiments. Field trials were planted with segregating hybrid seed from a single T_0_ event to produce large quantities of trait positive and null segregant (that is, transgene negative) senescent corn stover leaf.

### Characterisation of corn stover leaf extracts containing recombinant cellulase

**Figure 1:**
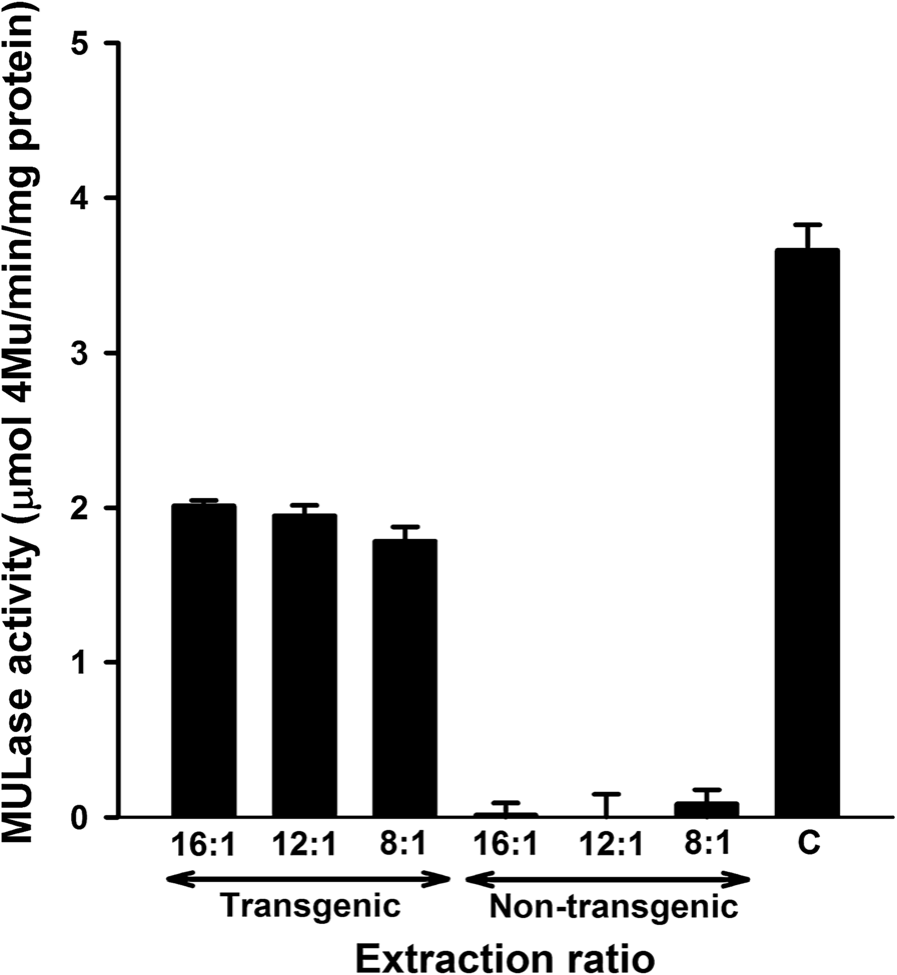
**Preparation and analysis of corn stover leaf extracts containing recombinant CBH**. Extracts were prepared at 16:1, 12:1, and 8:1 buffer-to-dry mass ratios. Extracts were prepared from non-transgenic corn stover leaf under the same conditions as negative controls. Cellulase activity was measured by monitoring the ability of extracts to release 4-methylumbelliferone (4-Mu) from 4-methylumbelliferyl-β-D-lactopyranoside (MUL) at pH 4.75 and 40°C, relative to a 4-Mu standard curve. Activity is presented as micromoles 4-Mu released per minute per milligram of protein and compared to the total MULase specific activity in Celluclast 1.5 L (C). All samples were analysed in triplicate, and error bars represent standard deviation.

Extracts from senescent, transgenic corn stover leaves were prepared at 16:1, 12:1, and 8:1 buffer-to-dry mass ratios, and cellulase activity in the extracts was measured using 4-methylumbelliferyl-β-D-lactopyranoside (MUL) as a substrate. Extracts were also prepared under identical conditions from non-transgenic corn stover leaf as controls. There was no significant difference between the MULase specific activity in the extracts prepared at 16:1 and 12:1 buffer-to-dry mass ratios (Figure 1). The extract obtained with the 16:1 buffer-to-dry mass ratio had a MULase specific activity that was about 55% that of the total MULase specific activity in Celluclast 1.5 L. There was, however, a 12% reduction in specific activity in the extract prepared at the 8:1 buffer-to-dry mass ratio (*P* < 0.05). It is likely that the reduction in MULase specific activity was caused by elevated concentrations of organic acids and/or phenolic compounds at the highest solids loading [36]. The MULase specific activities in protein extracts from non-transgenic corn events were minimal (<1%) relative to those measured in the extracts from transgenic events but did increase in the most concentrated non-transgenic extract to 5% of that obtained from the corresponding transgenic extract.

**Figure 2:**
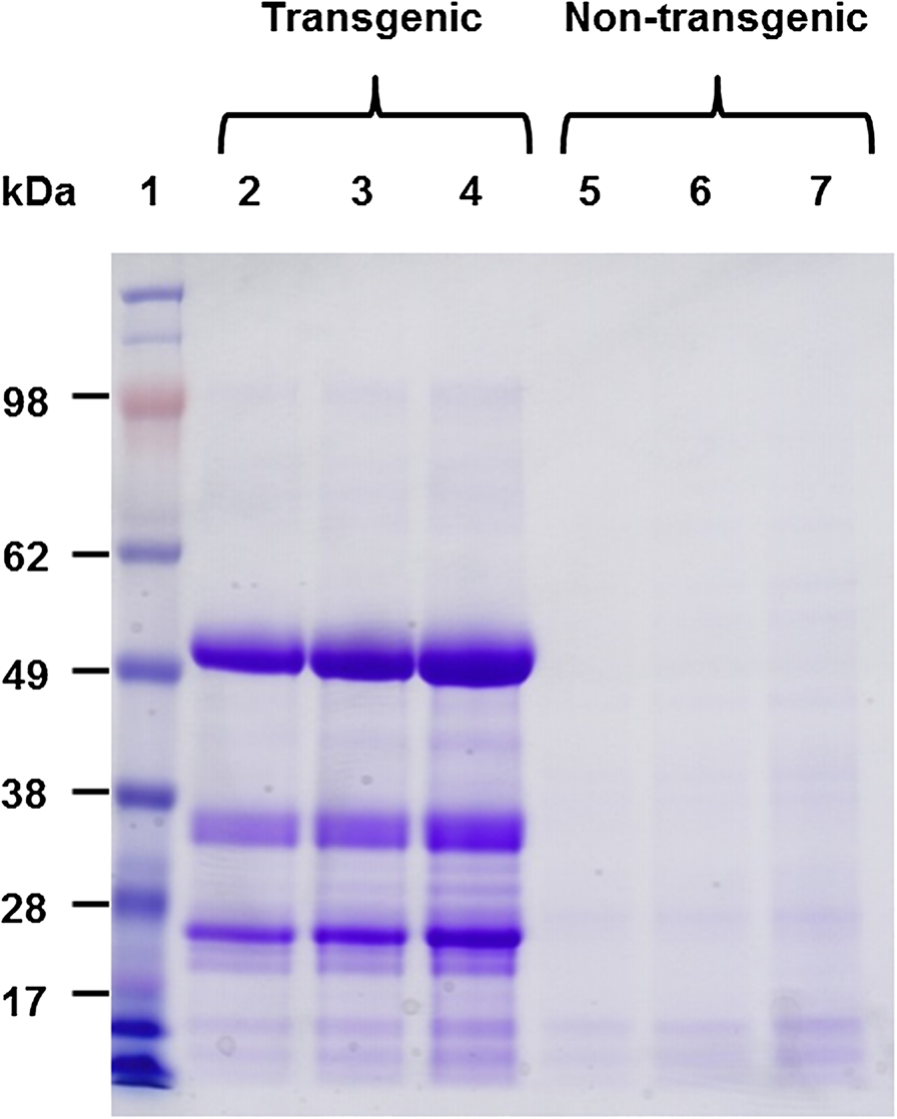
**SDS**-**PAGE analysis of corn stover leaf extracts**. Transgenic corn stover leaf extracts prepared at 16:1 (lane 2), 12:1 (lane 3), and 8:1 (lane 4) buffer-to-dry mass ratios were resolved using NuPAGE® 4-16% Bis-Tris gel (Invitrogen) with MES SDS buffer. Extracts from non-transgenic corn stover leaf prepared under corresponding conditions (lanes 5-7) were resolved as negative controls. SeeBlue® Plus2 (Invitrogen) was used as the size standard (lane 1). Equal volumes (10 μL) of extracts were analysed.

The apparent mass of the most abundant protein in the extracts from transgenic corn stover leaf was 52 kDa (Figure 2), which was similar to the predicted mass of recombinant CBH (53.3 kDa) based on the amino acid sequence of the mature protein [37]. The intensities of the 52-kDa band in the samples prepared at 16:1, 12:1, and 8:1 buffer-to-dry mass ratios correlated with MULase activity in the extracts (1.2, 1.6, and 2.4 U/mL, respectively). Minor bands were detected at apparent masses of 37 kDa and 27 kDa (Figure 2). The predicted mass of the catalytic domain of recombinant CBH is 45.4 kDa; therefore, it is unlikely that either band consisted of an intact CBH catalytic domain. There was no visible evidence of either the major band or two minor bands in the extracts from non-transgenic corn stover leaf.

### Pretreatment and characterisation of bagasse

Composition, particle size, crystallinity, the extent of defibrillation of the fibre bundles, and the nature of structural linkages are some of the parameters that influence the rate and extent of enzymatic hydrolysis of pretreated biomass. The compositions of untreated bagasse and bagasse pretreated in the Mackay Renewable Biocommodities Pilot Plant with dilute H_2_SO_4_ steam explosion, dilute NaOH steam explosion, and glycerol/HCl steam explosion under conditions described in our previous study [23] are presented in Table 1. The compositions of untreated (depithed) and bagasse pretreated at the laboratory scale with acidified EC/EG [38], BMIMCl [39], and ball-milling are presented in the same table. Percentages of glucan (cellulose), xylan (the majority of hemicellulose), lignin, and ash were calculated on a dry mass basis. Bagasse samples pretreated at the pilot scale were originally obtained from a sugar mill and contained dust, soil, and other debris accumulated during harvest, transport, and storage after juice extraction, leading to a high ash content in bagasse (Table 1). All pilot scale pretreatments increased the glucan content, with biomass pretreated with dilute NaOH having the highest content. The lowest lignin content in residue from the pilot scale pretreatment was achieved with dilute NaOH followed by acid-catalysed glycerol pretreatment, whereas xylan was almost completely removed during the dilute acid-catalysed, pilot scale pretreatments. At the laboratory scale, BMIMCl pretreatment and ball-milling did not obviously change the bagasse biomass composition, whereas acid-catalysed EC/EG pretreatment resulted in a significant increase in the glucan content and decreases in both lignin and xylan contents in the resulting fibre.

**Table 1:** **Biomass composition of pretreated sugar cane bagasse**

**Biomass**	**% Glucan**	**% Lignin**	**% Xylan**	**% Ash**	**CrI**
Untreated^*^	33.2	24.0	21.3	12.6	70
H_2_SO_4_ and steam explosion^*^	55.1	36.1	–	8.6	83
NaOH and steam explosion^*^	68.7	6.8	21.1	3.0	85
Glycerol/HCl and steam explosion^*^	57.0	25.7	–	12.9	82
Untreated (depithed)	42.8	26.3	27.2	5.1	74
Acidified EC/EG	77.0	6.1	8.9	–	78
BMIMCl	43.1	27.8	20.9	0.8	35
Ball-milling	42.7	26.9	24.1	1.2	14

– Not detected.

* Pilot scale pretreatments, reprinted from [23].

Scanning electron microscopy (SEM), Fourier transform infrared (FTIR) spectroscopy, and X-ray diffraction (XRD) were used to further characterise the pretreated bagasse. The results of these analyses for bagasse samples prepared from pilot scale processes were presented in our previous study [23]; only the results for bagasse samples pretreated at the laboratory scale using acidified EC/EG, BMIMCl, and ball-milling are presented herein. The typical diameter range of untreated (depithed) bagasse was 50 to 250 μm (Additional file 2: Figure S2a). Significant particle size reduction was observed after all laboratory scale pretreatments (Additional file 2: Figure S2b,c,d). The morphology of pretreated bagasse varied: acidified EC/EG pretreatment produced a large portion of defibrillated short fibres (with a diameter range of 15 to 30 μm) compared to defibrillated long fibres from NaOH pretreatment [23], and BMIMCl pretreatment produced irregular particles with diameters from 15 to 60 μm, while ball-milling produced a large portion of roughly spherical particles with a diameter range of 7 to 25 μm. The morphology of bagasse pretreated using EC/EG was consistent with that of our previous analyses [38].

A number of characteristic FTIR spectral features were used to monitor the chemical changes that occurred in lignin and carbohydrates in bagasse after pretreatment (Additional file 3: Figure S3). The intensities of lignin-associated peaks at 1728, 1630, 1599, 1512, 1464, 1416, 1329, 1244, and 835 cm^-1^ [23] all diminished significantly in the FTIR spectra of bagasse pretreated using acidified EC/EG (Additional file 3: Figure S3b), consistent with the removal of lignin (Table 1). All of the lignin-associated peaks in the FTIR spectra of bagasse pretreated using BMIMCl and ball-milling were still as prominent as those in untreated bagasse (Additional file 3: Figure S3c and d). The region of the FTIR spectrum of plant biomass from 1200 to 1000 cm^-1^ corresponds to C-O stretch and deformation in cellulose, lignin, and residual hemicellulose [40]. All pretreatments reduced absorbance at 1180 cm^-1^, with acidified EC/EG pretreatment reducing this feature most significantly. Absorbance at 1132 cm^-1^, which arises from crystalline cellulose [41], decreased after pretreatment with acidified EC/EG, while bagasse pretreated using BMIMCL and ball-milling had reduced absorbance at 1107 cm^-1^. Acidified EC/EG pretreatment significantly reduced absorbance at 1084 cm^-1^, but the feature was unchanged in residues from BMIMCl pretreatment and ball-milling. The peak at 898 cm^-1^ was assigned to C-O deformation in cellulose [42] and was more prominent in samples pretreated with acidified EC/EG, reflecting the relatively high glucan content (Table 1).

Five peaks in the XRD spectrum of cellulose (at 2θ = approximately 15°, 16°, 21°, 22.5°, and 34.5°) have been assigned to the crystalline form [43]. BMIMCl pretreatment and ball-milling reduced the resolution of the peaks at 2θ = 21°, 22.5°, and 34.5° (Additional file 4: Figure S4), suggesting that a significant proportion of the cellulose remaining after pretreatment was amorphous. The peaks observed in the XRD spectra of untreated and pretreated bagasse between 2θ at about 26° and 29° most likely arose from the presence of quartz and kaolinite, respectively, from soil contamination [44]. The untreated bagasse was obtained directly from a sugar mill, and soil typically accounts for about 1% of the wet mass of billeted cane. The CrI was estimated using the peak height method [45] (Table 1). BMIMCl pretreatment and ball-milling both reduced the cellulose crystallinity. All the other pretreatments increased the cellulose crystallinity because of the removal of amorphous components such as lignin, hemicellulose, and amorphous cellulose. A summary of the structural and compositional features of pretreated bagasse residues is presented in Table 2.

**Table 2:** **Changes in structural and compositional features of sugar cane bagasse after pretreatment analysed by spectroscopic methods**

**Biomass**	**Particle size**	**Defibrillation**	**Glucan content**	**Lignin content**	**Xylan content**	**Crystallinity**
H_2_SO_4_ and steam explosion^*^	↓	-	↑	↑	↓↓	↑
NaOH and steam explosion^*^	↓	↑↑	↑↑	↓↓	-	↑
Glycerol/HCl and steam explosion^*^	↓	↑	↑	-	↓↓	↑
Acidified EC/EG	↓	↑↑	↑↑	↓↓	↓↓	↑
BMIMCl	↓	-	-	-	-	↓↓
Ball-milling	↓↓	-	-	-	-	↓↓

↑, Increase; ↑↑, strong increase; −, no effect; ↓, decrease; ↓↓, strong decrease.

* Pilot scale pretreatments, reprinted from [23].

### Significant enhancement in enzymatic hydrolysis of bagasse pretreated with dilute H_2_SO_4_ steam explosion by adding corn stover-expressed CBH to Celluclast 1.5 L

**Figure 3:**
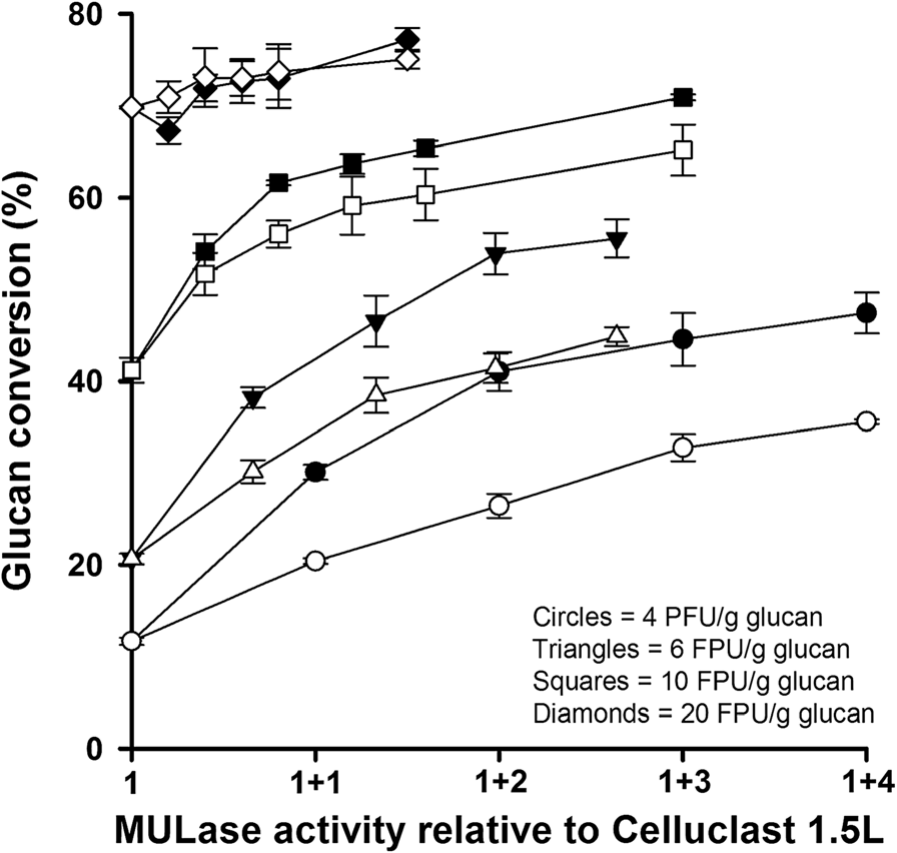
**Saccharification of H**_**2**_ **SO**_**4**_ **steam exploded bagasse by mixtures of Celluclast 1.5 L and corn stover leaf extracts**. Celluclast 1.5 L at dosages of 4 (circles), 6 (triangles), 10 (squares), and 20 (diamonds) FPU/g glucan were supplemented with MULase activity from transgenic corn stover leaf extracts containing recombinant CBH (black symbols) and used to saccharify H_2_SO_4_ steam exploded bagasse for 24 h. The numeral 1on the *x*-axis represents the total MULase activity present in the indicated dosage of Celluclast 1.5 L. Values above 1 indicate the addition of corn stover leaf extract containing recombinant CBH to Celluclast 1.5 L in units of MULase activity equal to the total MULase activity in Celluclast 1.5 L at each of the indicated dosages. Each reaction was supplemented with 50 μg β-glucosidase/g glucan. Equal volumes of extracts from non-transgenic corn stover leaves prepared under the same conditions were assessed for comparison (open symbols). Glucose release from cellulose was monitored using a colorimetric (GOPOD Format) assay and the results reported as the percentage of glucan converted to glucose. All samples were analysed in triplicate, and error bars represent standard deviation.

We previously demonstrated that dilute H_2_SO_4_ steam exploded bagasse was relatively resistant to enzymatic hydrolysis by commercial cellulase mixtures, when compared to bagasse pretreated with dilute NaOH steam explosion or glycerol/HCl steam explosion [23]. Therefore, we assessed the ability of plant-expressed CBH to enhance the performance of a commercial cellulase mixture on this substrate. Celluclast 1.5 L at a dosage of 4 filter paper units (FPU)/g glucan catalysed the conversion of 12% of the glucan in dilute H_2_SO_4_ steam exploded bagasse to glucose in 24 h (Figure 3). We then added increasing amounts of corn stover leaf extract containing recombinant CBH prepared at a 16:1 buffer-to-dry mass ratio and measured the effect on glucan conversion (Figure 3). Corn stover leaf extracts were added on the basis of MULase activity relative to the total MULase activity in Celluclast 1.5 L at a dosage of 4 FPU/g glucan. Therefore, the addition of an equal amount of MULase activity from transgenic corn stover leaf extract containing recombinant CBH resulted in a doubling of the total MULase activity in the reaction (1 + 1 on the *x*-axis of Figure 3), and so on. Equal volumes of extracts from the same mass of non-transgenic corn stover leaf were added as negative controls.

Recombinant CBH from corn stover leaf was able to enhance the performance of Celluclast 1.5 L to a maximum of 45% glucan conversion at a total of 4 added units of MULase activity (Figure 3). In contrast, extract from non-transgenic corn stover leaf enhanced the performance of Celluclast 1.5 L to a maximum of 30% glucan conversion. A comparison of the results obtained from transgenic and non-transgenic corn stover leaf extracts (Figure 3) demonstrated that the absolute improvement in Celluclast 1.5 L performance from the addition of increased amounts of recombinant CBH activity ranged from 9 to 15% glucan conversion, although the relative contribution from the transgenic extract remained constant at about 33% of the total glucan conversion.

To determine if the additive function of corn stover leaf extract containing recombinant CBH was affected by changes in the buffer-to-dry mass ratio employed during extraction, we compared the ability of corn stover leaf extracts from transgenic events expressing CBH and non-transgenic events prepared at 16:1, 12:1, and 8:1 buffer-to-dry mass ratios to enhance the performance of Celluclast 1.5 L at a dosage of 4 FPU/g glucan (Additional file 5: Figure S5). All of the extracts prepared from transgenic corn stover leaf expressing recombinant CBH were able to enhance the performance of Celluclast 1.5 L at a dosage of 4 FPU/g glucan. Extracts from non-transgenic corn were also able to enhance the performance of Celluclast 1.5 L, but to a significantly lower level than extracts from transgenic corn. Importantly, the buffer-to-dry mass ratio used for extraction did not appear to have a significant impact on the ability of the recombinant CBH to enhance the performance of Celluclast 1.5 L. Therefore, we used recombinant CBH extracts prepared at 16:1, 12:1, and 8:1 buffer-to-dry mass ratios as additives to Celluclast 1.5 L at increased dosages (6, 10, and 20 FPU/g glucan, Figure 3). The results of these analyses indicated that corn stover leaf extracts containing recombinant CBH significantly enhanced the performance of Celluclast 1.5 L on dilute H_2_SO_4_ steam exploded bagasse at dosages of 6 and 10 FPU/g glucan but not at a dosage of 20 FPU/g glucan.

### Effect of addition of corn stover-expressed CBH to Cellic CTec2 on hydrolysis of bagasse pretreated with different processes

**Figure 4:**
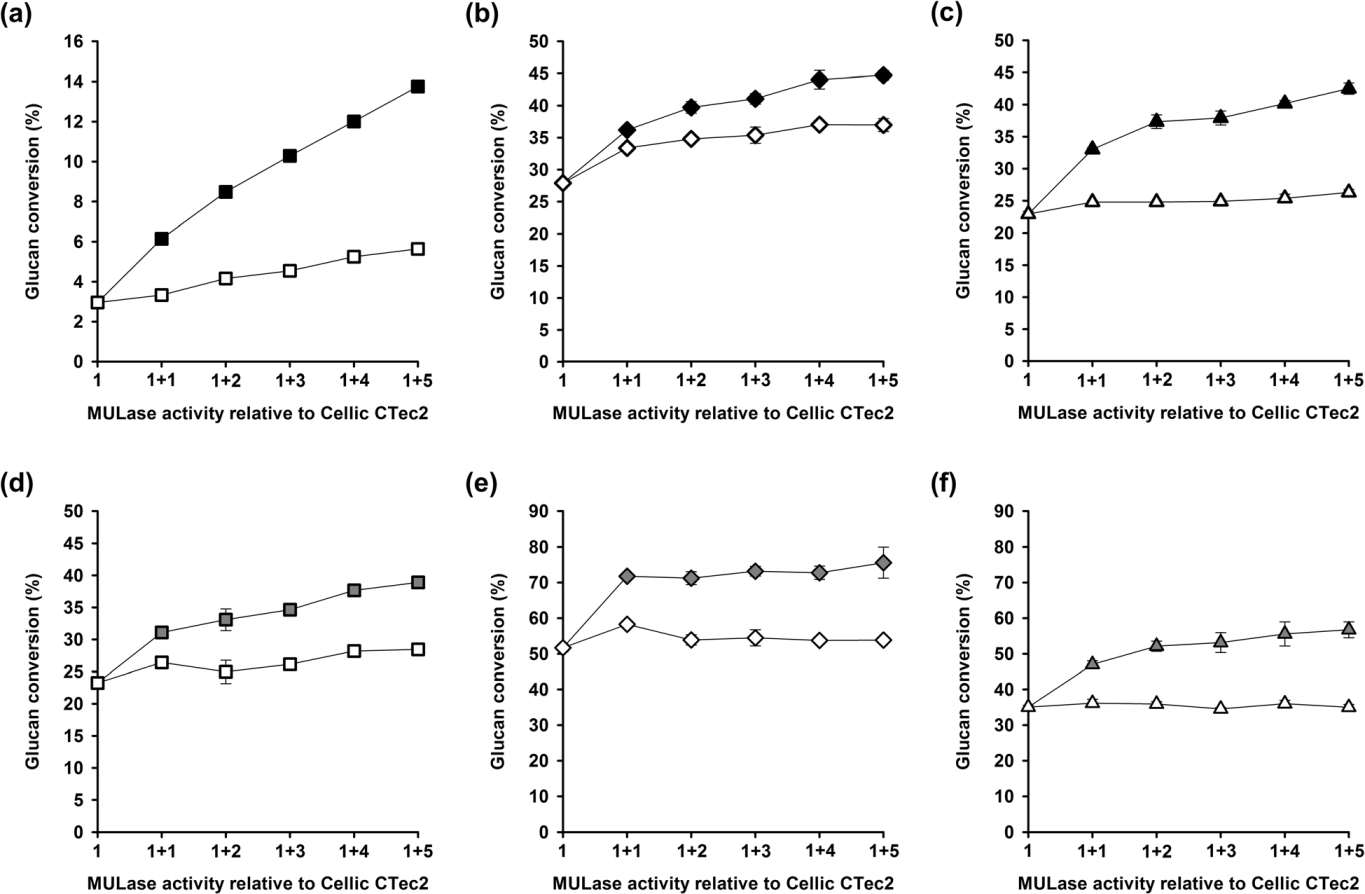
**Saccharification of pretreated bagasse by mixtures of Cellic CTec2 and transgenic corn stover leaf extracts**. **(a)** H_2_SO_4_ steam explosion. **(b)** NaOH steam explosion. **(c)** Glycerol/HCl steam explosion. **(d)** EC/EG. **(e)** BMIMCl. **(f)** Ball-milling. Cellic CTec2 at a dosage of 2 FPU/g glucan was supplemented with MULase activity from transgenic corn stover leaf extract (black symbols) and used to saccharify pretreated bagasse for 24 h. Each unit on the *x*-axis represents the total MULase activity present in Cellic CTec2. Values above 1 indicate the addition of MULase activity from transgenic corn stover leaf extract (black symbols) to Cellic CTec2. Each additional unit of MULase activity supplied from transgenic corn stover leaf extract containing CBH is equal to the total MULase activity in Cellic CTec2 at a dosage of 2 FPU/g glucan. Each reaction was supplemented with 50 μg β-glucosidase/g glucan. Equal volumes of extracts from non-transgenic corn stover leaves prepared under the same conditions were assessed for comparison (open symbols). Glucose release from cellulose was monitored using a colorimetric (GOPOD) assay and the results reported as the percentage of glucan converted to glucose. All samples were analysed in triplicate, and error bars represent standard deviation.

Celluclast 1.5 L (Novozymes) has been available for at least 20 years and represents first generation fibrolytic technology. Cellic CTec2 (Novozymes) is a commercial cellulase mixture that was released in 2010 and has been used in subsequent experiments. The ability of corn stover leaf-expressed CBH to enhance the performance of Cellic CTec2 at a dosage of 2 FPU/g glucan was assessed using the six pretreated bagasse substrates (Figure 4). As described in the previous section, transgenic corn stover leaf extracts were added on the basis of MULase activity relative to the total MULase activity in Cellic CTec2 at a dosage of 2 FPU/g glucan. Protein extracts from non-transgenic corn stover were included as negative controls. We observed that bagasse samples with significantly decreased cellulose crystallinity were hydrolysed more readily by Cellic CTec2. Further, corn stover leaf-expressed CBH enhanced the performance of Cellic CTec2 to a significantly higher level than extracts from non-transgenic corn stover leaf for all pretreatments. The absolute CBH-dependent increases in glucan conversion after 24 h are presented in Table 3. The Cellic CTec2 dose responses on bagasse pretreated using dilute H_2_SO_4_ steam explosion, dilute NaOH steam explosion, glycerol/HCl steam explosion, acidified EC/EG, BMIMCl, and ball-milling were determined (Additional file 6: Figure S6), thereby allowing estimation of the Cellic CTec2 dosage (FPU/g glucan) equivalent to 2 FPU/g glucan supplemented with 5 units of MULase activity from transgenic corn stover leaf extract containing recombinant CBH (1 + 5, Table 3). The overall performance of the mixture improved by a minimum of 50% (that is, equivalent to Cellic CTec2 at 3 FPU/g glucan) to a maximum of 3.5-fold (equivalent to Cellic CTec2 at 7 FPU/g glucan) for glycerol/HCl steam-exploded bagasse.

**Table 3:** **Enhancement of Cellic CTec2 at a loading of 2 FPU**/**g glucan on pretreated sugar cane bagasse by recombinant CBH in transgenic corn stover leaf extract**

**Pretreatment**	**Recombinant CBH-dependent increase in % glucan conversion**	**Cellic CTec2 dosage equivalent to Cellic CTec2 at 2 FPU/g glucan plus 5 units recombinant CBH**
H_2_SO_4_ and steam explosion	8.1	6
NaOH and steam explosion	7.8	3
Glycerol/HCl and steam explosion	16.2	7
Acidified EC/EG	10.5	3
BMIMCl	21.8	6
Ball-milling	21.7	5

**Figure 5:**
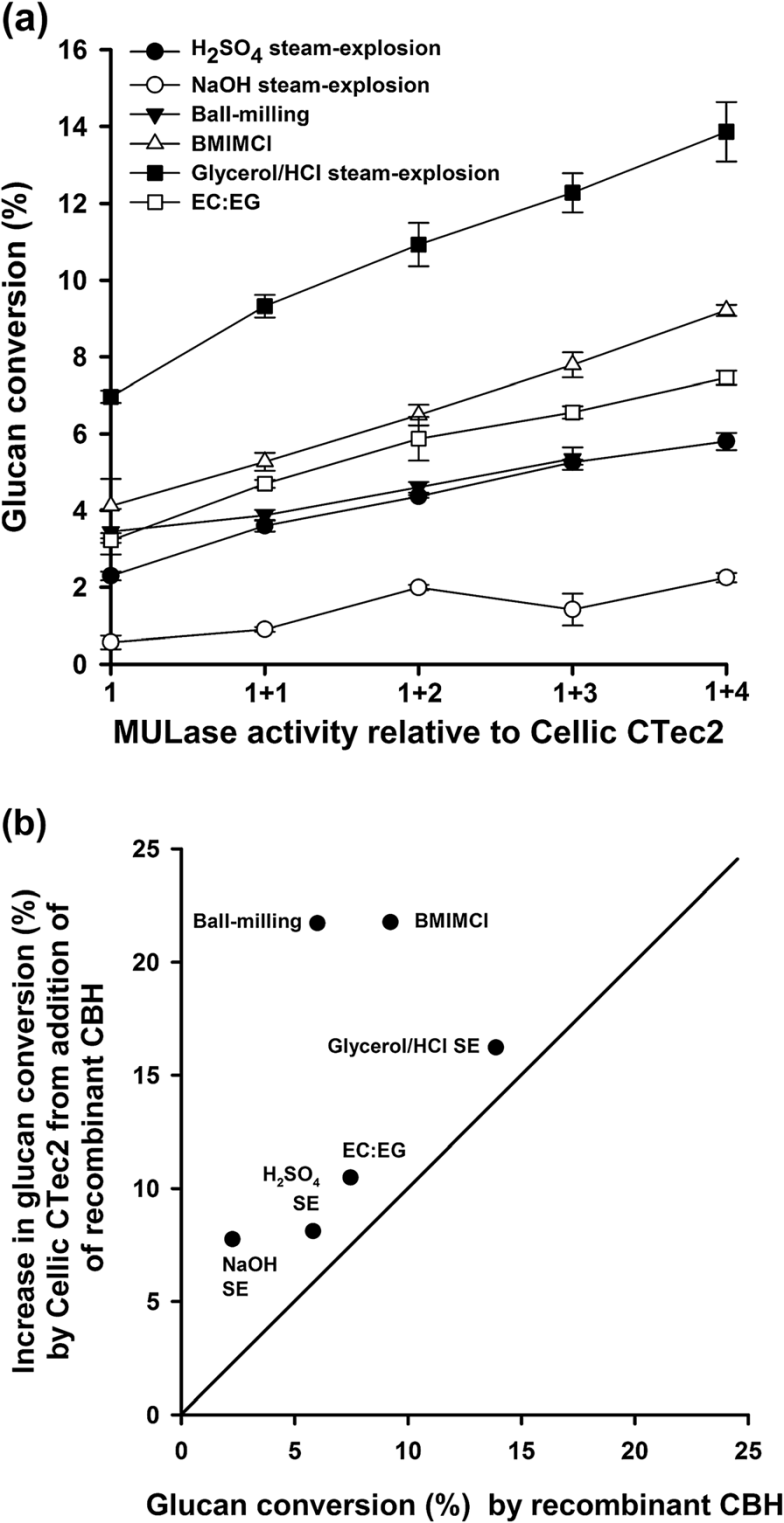
**Saccharification of pretreated bagasse by transgenic corn stover leaf extracts containing recombinant CBH**. **(a)** Pretreated bagasse samples were saccharified for 24 h with extracts from transgenic corn stover leaf containing recombinant CBH. The numerals on the *x*-axis represent the total loading of MULase activity from transgenic corn stover leaf extract, in multiples of the total MULase activity present in Cellic CTec2 at a dosage of 2 FPU/g glucan. The transgenic-extract MULase loadings designated as “1 + 4” in this figure are each thus equal to the loadings of “additional” transgenic-extract MULase activity added to Cellic CTec2 in the loading designated as “1 + 5” in Figure 4. Each reaction was supplemented with 50 μg β-glucosidase/g glucan. Glucose release from glucan was monitored using a colorimetric (GOPOD) assay and the results reported as the percentage of glucan converted to glucose. All samples were analysed in triplicate, and error bars represent standard deviation. **(b)** Comparison between the ability of corn stover leaf extract containing recombinant CBH to hydrolyse pretreated bagasse and its ability to enhance the performance of a commercial cellulase mixture. The black line indicates equal performance alone and in combination with a commercial cellulase mixture.

To more fully understand the impact of plant-expressed CBH on the performance of the commercial cellulase mixture, we assessed the ability of plant-expressed CBH to hydrolyse pretreated sugar cane bagasse (Figure 5a) at the same MULase activities used to enhance the performance of Cellic CTec2 (Figure 4). Glycerol/HCl steam exploded bagasse was the most susceptible to degradation by plant-expressed CBH, with a maximum glucan conversion of 14%. The susceptibilities of bagasse pretreated using dilute H_2_SO_4_ steam explosion acidified EC/EG, BMIMCl, and ball-milling to hydrolysis were similar and ranged from 5 to 7% glucan conversion. Dilute NaOH steam exploded bagasse was the least susceptible to hydrolysis by plant-expressed CBH, with a maximum glucan conversion of about 2% (Figure 5a). We have previously shown that glycerol/HCl steam exploded bagasse was more susceptible to hydrolysis by microbially expressed CBH than dilute H_2_SO_4_ steam exploded and dilute NaOH steam exploded bagasse, in that order [23]. Comparison with the results obtained for mixtures of Cellic CTec2 and the same dosages of plant-expressed CBH (Figure 4) revealed that the ability of plant-expressed CBH to enhance the performance of Cellic CTec2 was greater than the ability of the enzyme to hydrolyse the substrates alone (Figure 5b). This was expected, because commercial cellulase mixtures contain enzymes (for example*,* EG, glycoside hydrolase 61) and other proteins (such as expansin-like proteins) that act synergistically with CBHs [46–49]. Interestingly, the ability of recombinant CBH expressed *in planta* to enhance the performance of Cellic CTec2 on ball-milled or BMIMCl pretreated bagasse relative to its ability to hydrolyse the substrate alone was significantly greater than on the remaining substrates.

**Figure 6:**
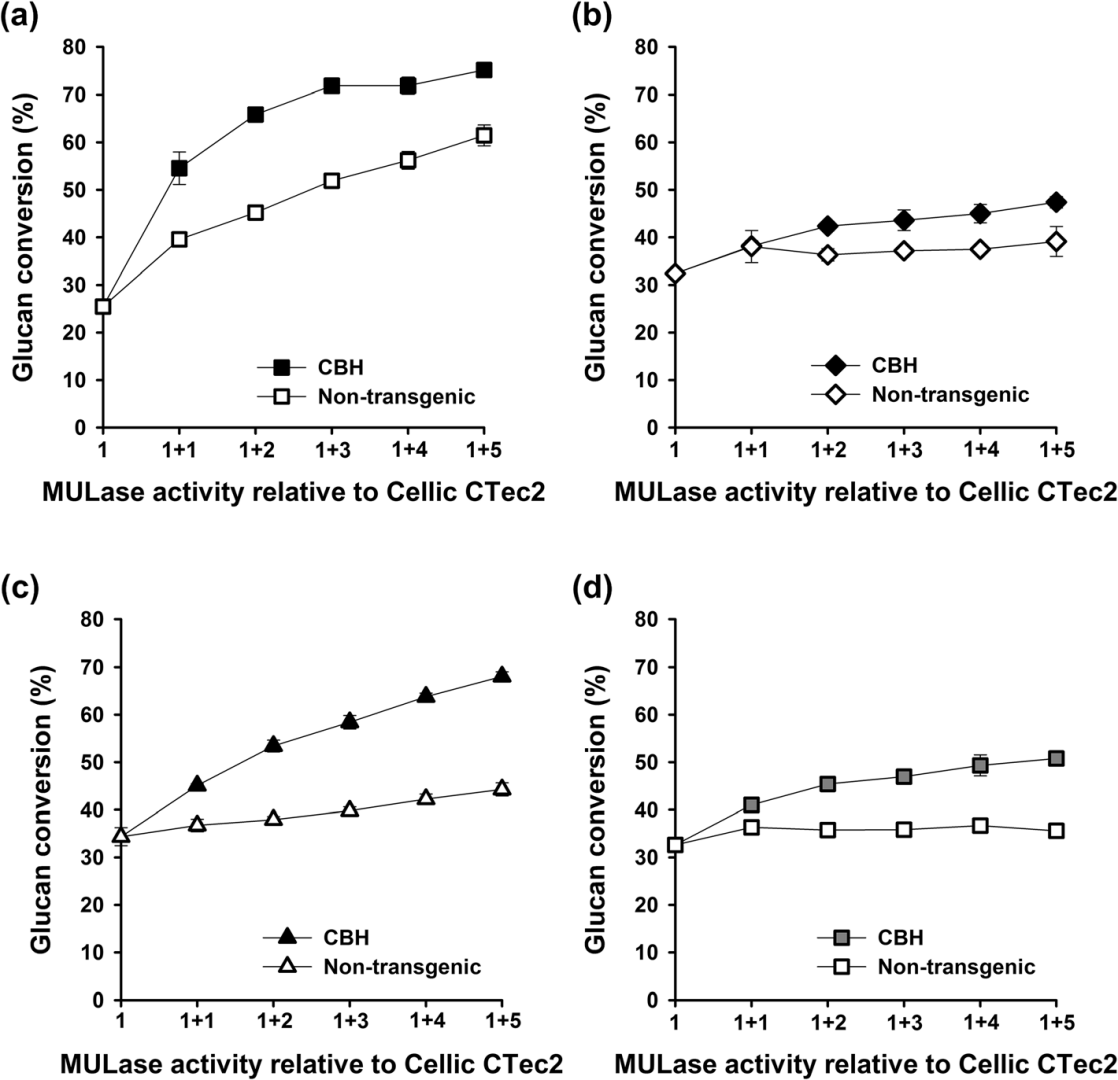
**Saccharification of pretreated bagasse with mixtures of Cellic CTec2 and transgenic corn stover leaf extract**. **(a)** H_2_SO_4_ steam explosion. **(b)** NaOH steam explosion. **(c)** Glycerol/HCl steam explosion. **(d)** EC/EG. Pretreated bagasse samples were saccharified with a dosage of Cellic CTec2 that resulted in about 30% glucan conversion after 24 h. Cellic CTec2 at these dosages was supplemented with MULase activity from transgenic corn stover leaf extract containing recombinant CBH (black symbols). Values above 1 indicate the addition of corn stover leaf extract containing recombinant CBH to Cellic CTec2 in units of MULase activity equal to the total MULase activity in Cellic CTec2 at each of the indicated dosages. Equal volumes of extracts from non-transgenic corn stover leaves prepared under the same conditions were assessed for comparison (open symbols).

Given the variation in cellulose crystallinity in the substrates (Table 1) and susceptibility to enzymatic hydrolysis by Cellic CTec2 (Additional file 6: Figure S6), it was possible that the glucan conversion (%) itself had an impact on the ability of corn stover leaf-expressed CBH to enhance the performance of Cellic CTec2 (Figure 4). Therefore, we selected a Cellic CTec2 dosage that would catalyse about 30% glucan conversion in dilute H_2_SO_4_ steam exploded, dilute NaOH steam exploded, glycerol/HCl steam exploded, and acidified EC/EG pretreated bagasse after 24 h (Table 4) and assessed the ability of corn stover leaf-expressed CBH to enhance Cellic CTec2 performance at those dosages (Figure 6). Extracts from an equivalent mass of non-transgenic corn stover were included as controls. The average glucan conversion by Cellic CTec2 at the selected dosages was 31.3 ± 3.9%. Corn stover leaf-expressed CBH enhanced the performance of Cellic CTec2 on all four substrates. Further, we estimated the Cellic CTec2 dosage required to give an equivalent performance to that of Cellic CTec2 at a dosage of 2 FPU/g glucan supplemented with 5 units of total MULase activity from transgenic corn stover leaf extract containing recombinant CBH (1 + 5, Table 4). The overall performance of the mixture improved from a minimum of twofold to a maximum of fourfold for bagasse pretreated using glycerol/HCl steam explosion.

**Table 4:** **Enhancement of Cellic CTec2 performance at a dosage resulting in** about **30**% **glucan conversion of pretreated sugar cane bagasse by recombinant CBH in transgenic corn stover leaf extract**

**Pretreatment**	**Cellic CTec2 dosage resulting in about 30% glucan conversion (FPU/g glucan)**	**Cellic CTec2 dosage equivalent to the performance of Cellic CTec2 plus 5 units recombinant CBH (FPU/g glucan)**	**Fold increase in Cellic CTec2 performance**
H_2_SO_4_ and steam explosion	9.0	25	3
NaOH and steam explosion	1.5	3	2
Glycerol/HCl and steam explosion	3.0	13	4
Acidified EC/EG	1.5	4	2

### Effect of disruption of transgenic corn stover leaves on recombinant cellobiohydrolase extraction in tap water

**Figure 7:**
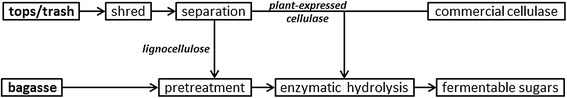
**Model for** “**mixed delivery**” **of plant**-**expressed cellulase into a commercial sugar cane enzymatic hydrolysis system**.

**Figure 8:**
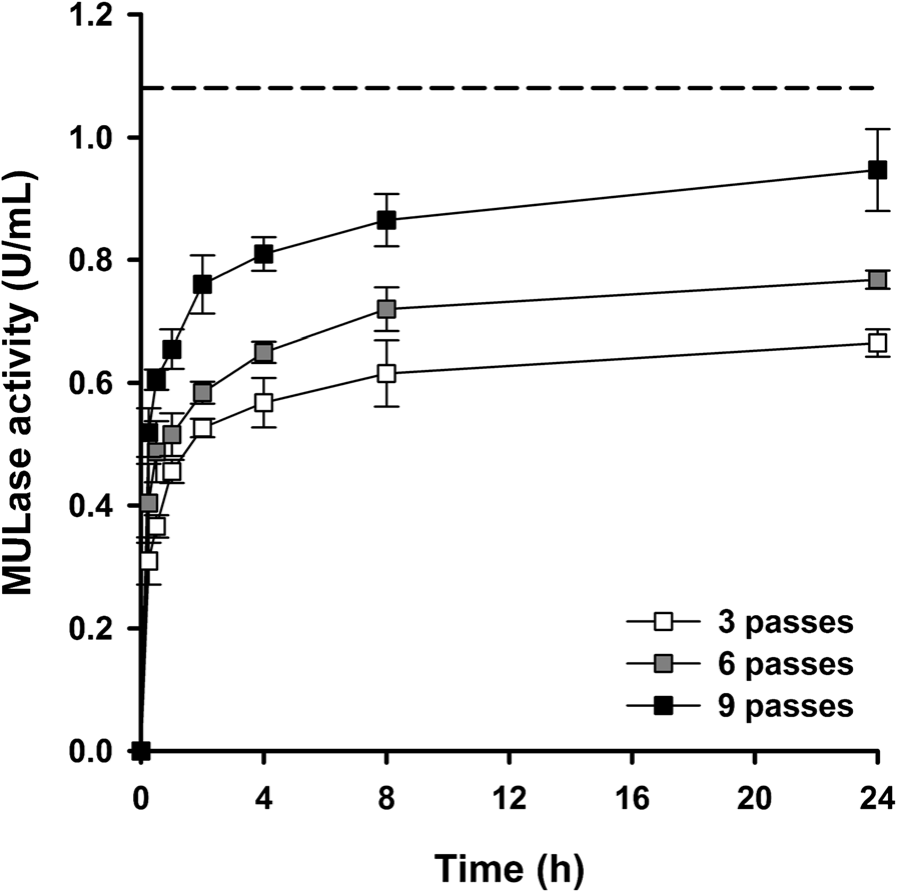
**Extraction of CBH from transgenic corn stover leaf into tap water after mild disruption**. Transgenic corn stover leaf containing recombinant CBH was disrupted with three (white squares), six (grey squares), or nine (black squares) passes through a cutting mill. Three water extracts were prepared for each level of tissue disruption. Water extracts were also prepared from corn stover leaf milled to a particle size of <2 mm and disrupted using a bead mill (dotted line). CBH activity was measured in the extracts by monitoring the ability of extracts to release 4-methylumbelliferone (4-Mu) from 4-methylumbelliferyl-β-D-lactopyranoside (MUL) at pH 4.75 and 40°C, relative to a 4-Mu standard curve. Activity is presented as micromoles 4-Mu released per min per mg of protein. All samples were analysed in triplicate, and error bars represent standard deviation.

Having demonstrated that recombinant CBH expressed in transgenic corn stover leaf enhanced the performance of a commercial cellulase mixture on a wide range of pretreated sugar cane bagasse substrates at the microtube scale, we explored a more industrially relevant process for deploying plant-expressed, recombinant CBH into a larger scale hydrolytic system. We envisaged a process in which transgenic corn stover leaf containing recombinant CBH was crudely shredded, extracted using tap water, and then resolved into an extract stream for addition to a commercial cellulase mixture for hydrolysis and a residual lignocellulose stream for pretreatment (Figure 7). Therefore, we assessed the extraction of CBH from transgenic corn stover leaf in water after crude shredding. Transgenic corn stover leaf was disrupted using three, six, or nine passes through a cutting mill to generate particles ranging in length from millimetres to centimetres (Additional file 7: Figure S7). Tap water was used to extract recombinant CBH from the crudely disrupted corn stover leaf at ambient temperature, and the MULase activity in solution was measured (Figure 8). Disruption of corn stover leaf using nine passes through the cutting mill resulted in the greatest release of recombinant CBH. Importantly, the maximum CBH release was 88% of that obtained from ball-milled corn stover leaf after 24 h, despite the leaf only being coarsely disrupted using a cutting mill. All three samples reached 85% of their maximum extracted MULase activity after 4 h.

## Discussion

Commercial cellulase mixtures derived from fungal cultures typically contain a complex mixture of enzymes that degrade cellulose (endoglucanase, exoglucanase, β-glucosidase, and copper-dependent oxygenase) and hemicellulose (xylanase, β-xylosidase, acetyl xylan esterase, arabinofuranosidase, β-mannanase, α-galactosidase, α-glucuronidase, and β-galactosidase), as well as proteins that enhance the performance of the other components (expansin-like proteins) [11]. The ability of recombinant CBH expressed in corn stover leaf to enhance the performance of Celluclast 1.5 L on dilute H_2_SO_4_ steam exploded sugar cane bagasse diminished with increasing dosage of the commercial cellulase mixture. This was expected, because CBH activity is rate-limiting for cellulose hydrolysis [50] and CBHs make up the majority of protein mass in commercial cellulase mixtures [13]. Further, we would expect the same to be true for sugar cane bagasse pretreated using the alkaline, green solvent, and physical processes described herein. We have previously shown that increasing the dosage of a commercial cellulase mixture above the optimum increased the rate with which maximum glucan conversion was achieved but did not increase maximum glucan conversion [23]. Given that glucan hydrolysis was measured at 24 h, it is possible that plant-expressed CBH enhanced the rate at which maximum glucan conversion was achieved by Celluclast 1.5 L at a dosage of 20 FPU/g glucan without increasing maximum glucan conversion.

There is a negative correlation (R^2^ = 0.86) between the lignin and glucan contents of sugar cane fibres pretreated using dilute H_2_SO_4_ steam explosion, dilute NaOH steam explosion, glycerol/HCl steam explosion, and acidified EC/EG (Table 1). These residues were subjected to enzymatic hydrolysis using dosages of a commercial cellulase mixture that resulted in an equivalent glucan conversion after 24 h, and the ability of both extracts from transgenic and non-transgenic corn stover leaf to enhance the performance of the commercial cellulase mixtures was assessed. Our results demonstrated that extracts from non-transgenic corn stover leaf were able to significantly enhance the performance of the commercial cellulase mixture. Interestingly, the magnitude of the enhancement was different for each pretreatment (3 to 36% glucan conversion), and there was a positive correlation (R^2^ = 0.76) between the lignin content in the residue and the magnitude of the enhancement. Protein extracts from non-transgenic corn seed, shoot, and stem have been shown to enhance the performance of commercial cellulase mixtures on a pure cellulosic substrate [51,52] with a crystallinity index between 53% and 91%, depending on the method used to determine crystallinity [43]. In contrast to these studies, the non-transgenic extracts generated in the present study did not contain appreciable MULase activity (Figure 1) or the ability to hydrolyse carboxymethylcellulose (data not shown), the substrates were pretreated lignocellulosic biomass, and glucan conversions were, with the exception of a single experimental condition, above 10%. Bovine serum albumin and corn stover hydrophobic proteins have also been shown to enhance the enzymatic hydrolysis of pretreated plant biomass by commercial cellulase mixtures [53–57] by binding to lignin in place of cellulases [58]. Our results are consistent with a mode of activity in which native corn stover leaf proteins bind to lignin in pretreated sugar cane residues and prevent cellulase binding.

Significant improvements in the enzymatic digestion of pretreated biomass with commercial cellulase mixtures are achievable with the addition of plant-expressed CBH extracted in a very simple buffer system without concentration. These improvements manifest economic benefits for a commercial production facility, including a reduction in commercial cellulase addition rates (decreasing production costs), a reduction in hydrolysis time (decreasing capital costs), an increase in glucose yield (increasing revenue), or some combination of these benefits. The extent of these economic benefits is dependent upon the relative digestibility of the pretreated biomass, cost, and effectiveness of the commercial cellulase mixture, the yield of plant-expressed cellulases, and the cost of extraction of plant-expressed cellulases. Our work has shown that highly effective pretreatment strategies such as glycerol/HCl are able to achieve benchmark performance targets of about 90% glucan conversion in 24 h with a cellulase addition rate of 20 FPU/g glucan compared to dilute H_2_SO_4_ steam explosion pretreatment, which achieves about 70% glucan conversion in 72 h at the same cellulase addition rate. The utilisation of plant-expressed CBH in addition to the commercial cellulase mixtures enabled a fourfold reduction in the commercial cellulase cocktail addition rate to glycerol/HCl pretreated bagasse for the same glucan conversion yield. Efficient extraction of recombinant CBH from transgenic corn stover leaf was achieved in a simple process using water after minimal tissue disruption.

## Conclusions

CBHs play a central role in the enzymatic hydrolysis of cellulose in lignocellulosic biomass, and we chose to express CBH in transgenic corn, rather than another cellulase or an accessory enzyme/protein. Recombinant CBH enhanced the performance of a commercial cellulase mixture on all pretreated bagasse residues tested. Taken together, our experimental results indicate the potential for substantial economic benefits from the integration of plant-expressed cellulases into a commercial facility producing fermentable sugars from lignocellulosic biomass, regardless of the pretreatment technology being employed.

## Methods

### Materials

Sugar cane bagasse, provided by Racecourse Sugar Mill (Mackay, Australia), was washed with copious amounts of water in the sugar mill to remove residual sugars. The sugar cane bagasse for the pilot scale pretreatment was delivered directly to the Mackay Renewable Biocommodities Pilot Plant. The sugar cane bagasse for laboratory-scale pretreatment was air-dried to constant mass and milled in a Retsch® SM 100 hammer mill (Retsch® GmBH, Germany). The milled bagasse was screened using sieves, and bagasse powder with particle sizes from 250 to 500 μm was collected and stored at room temperature (24°C) in a sealed container. The moisture content of the bagasse powder was 6.9%. Celluclast 1.5 L (batch number 041M1810V), a Novozymes product, was purchased through Sigma. Cellic CTec2 (batch number VCPI0003), a Novozymes product, was supplied by Novozymes to Syngenta Biotechnology Inc. and the Queensland University of Technology (QUT). *Aspergillus niger* β-glucosidase solution (lot number 90701) was purchased from Megazyme (Ireland).

### Cellulase expression in transgenic corn

The CBH utilised in this study (Additional file 1: Figure S1) is a proprietary biomolecule obtained from Verenium Corporation, San Diego, CA, USA [32]. The construction of the expression cassette used to transform corn (*Zm*-*PepC*-CBH I-VSD) has been described previously [37]. The CBH utilised in this study shares 96% amino acid sequence identity with CBH I from *Penicillium occitanis* [33] and 61% amino acid sequence identity with CBH I (Cel7A) from *Trichoderma reesei* [34].

The corn (*Zea mays* inbred variety AX5707) embryogenic callus induction, *Agrobacterium*-mediated transformation, and regeneration of transgenic corn were performed as described previously [59]. Transgenic events were analysed using TaqMan real-time PCR analysis following the methods described by Ingham *et al*. [60] for the presence of the transgene and selectable marker, and transferred to the glasshouse. Transgenic plants were hybridised with corn variety ID5829 for seed production. Subsequent generations were monitored for the presence and copy number of the transgene using TaqMan real-time PCR [60]. Transgenic plants were grown in the Syngenta Biotechnology Inc. continuous nursery in Kauai, Hawaii, to generate true corn stover leaf. Field senesced leaves were collected one week prior to seed harvest and pooled from heterozygous and homozygous plants into a single batch. To minimise colonisation by microorganisms, the corn stover leaf was air-dried for about three days. Transgene negative field senesced leaves to provide control material for laboratory studies were harvested and pooled.

### Preparation and analysis of corn stover leaf extracts

Corn stover leaf for microplate hydrolysis experiments was disrupted using the Retsch hammer mill fitted with a 2 mm retention screen. Particles of < 2 mm were collected and freeze dried. Extracts were prepared using aliquots (2 g) of dry corn stover leaf and extraction buffer (100 mM sodium acetate pH 4.75, 0.02% (w/v) sodium azide) at 16:1, 12:1, and 8:1 buffer-to-dry mass ratios. Extraction was allowed to proceed for 1 h at 4°C with mixing by inversion. Extracts were clarified by centrifugation at 4,750 × *g* (Allegra X-15R, Beckman Coulter, USA) and stored at 4°C until required.

Corn stover leaf for the tap water extraction experiments was disrupted using the cutting mill (Retsch, SM 100). With the retention screen removed, corn stover leaf was passed three, six, or nine times through the mill. Extracts were generated using tap water (containing 0.02% (w/v) sodium azide) as the solvent and a solids loading of 5% (w/w). Extraction was performed in sealed conical flasks at ambient temperature (about 23°C) with mixing by shaking at 150 rpm. Control extracts were prepared by disruption of corn stover leaf through the cutting mill fitted with a 2-mm retention screen. Particles of <2 mm were collected, further disrupted using a bead-beater (QIAGEN, TissueLyser2, USA), and extracted in 2-mL microcentrifuge tubes under the conditions described above.

The soluble protein concentration was measured using the Bradford assay (Bio-Rad Protein Assay, Bio-Rad, USA). As described previously [37], the cellulase activity was measured by monitoring the ability of protein extracts to release fluorescent 4-methylumbelliferone (4-Mu) from 0.91 mM 4-methylumbelliferyl-β-D-lactopyranoside (MUL) [61] in the presence of 13.46 mM δ-gluconolactone after 20 min at 40°C and pH 4.75, relative to a 4-Mu standard curve. The fluorescence was measured using an LS 50B Luminescence Spectrometer (Perkin Elmer, Glen Waverley, Victoria, Australia). The 4-Mu, MUL, and δ-gluconolactone were supplied by Sigma-Aldrich (Sydney, Australia). δ-gluconolactone was added to inhibit the hydrolysis of MUL by β-glucosidases [62]. The cellulase activity measured in commercial cellulase mixtures produced by native or transgenic *Trichoderma reesei* using MUL as a substrate is a combination of the activities of CBH I, CBH II, and EG I [62], and is therefore designated herein as “total MULase” activity. Protein extracts were resolved by sodium dodecyl sulphate-polyacrylamide gel electrophoresis (SDS-PAGE) using NuPAGE® 4-16% Bis-Tris gels (Invitrogen, Australia). SeeBlue® Plus2 (Invitrogen, Australia) was used as the protein size standard.

### Pretreatment of sugar cane bagasse

We have previously described the pilot scale pretreatment of sugar cane bagasse using H_2_SO_4_ steam explosion, NaOH steam explosion, and glycerol/HCl steam explosion [23]. To maintain consistency, the same batches of sugar cane bagasse pretreated using these methods were used in the present study. To expand the available range of pretreated sugar cane bagasse, air-dried and depithed sugar cane bagasse was pretreated at the laboratory scale using a mixture of EC and EG, an ionic liquid (BMIMCl), or a purely physical pretreatment (ball-milling). The EC/EG pretreatment was undertaken at a solid-to-liquid ratio of 4:1 (w/w) in the presence of 1.2% (w/w) H_2_SO_4_ at 90°C for 30 min using the method described by Zhang *et al.* (2013) [38]. The ionic liquid pretreatment was undertaken with BMIMCl containing 0.7% (w/w) water at a 10:1 (w/w) liquid-to-solid ratio at 150°C for 1 h using the method described by Karatzos *et al.* (2012) [39]. Ball-milling was achieved using a Pulverisette 6 (Fritsch, GmbH) at ambient temperature using 10 min on/off for a total of 16 h. All pretreated bagasse samples were washed extensively with deionised water and freeze dried. The aggregated fibre particles were separated in a mortar and pestle to detach large (>0.5 mm) particles, sieved, and particles ≤ 0.25 mm were collected for further analysis. Particles larger than 0.25 mm were gently ground in a mortar and pestle and re-sieved. Untreated bagasse was ground and sieved using the same procedure. Compositional analyses of sieved bagasse and sieved pretreated bagasse were conducted according to a standard procedure developed by the National Renewable Energy Laboratory (NREL, USA) [63].

### Characterisation of untreated and pretreated sugar cane bagasse

Untreated bagasse and pretreated bagasse samples were characterised by XRD, SEM, and FTIR spectroscopy using the methods described previously [23].

### Enzymatic hydrolysis of pretreated sugar cane bagasse

Enzymatic hydrolysis of sugar cane bagasse at the microtube scale was undertaken as described previously [23]. Commercial cellulase mixtures were desalted and buffer-exchanged as described therein. Corn-expressed CBH loadings corresponded to the addition of 2- to 20-fold the total MULase activity present in Cellic CTec2 at a dosage of 2 FPU/g glucan.

## Additional files

Additional file 1: Figure S1. Amino acid sequence of recombinant CBH. The N-terminal signal peptide is underlined. The C-terminal vacuolar sorting determinant is double-underlined. Additional file 2: Figure S2. SEM analysis. (a) Untreated sugar cane bagasse and sugar cane bagasse pretreated with (b) acidified EC/EG, (c) BMIMCl, and (d) ball-milling. Additional file 3: Figure S3. FTIR analysis. (a) FTIR spectra of untreated sugar cane bagasse and comparison with sugar cane bagasse pretreated with acidified EC/EG, BMIMCl, and ball-milling. FTIR difference spectra between untreated (depithed) bagasse and bagasse pretreated with (b) acidified EC/EG, (c) BMIMCl, and (d) ball-milling. A positive value in the FTIR difference spectra corresponds to the loss of a spectral feature relative to untreated bagasse. Additional file 4: Figure S4. XRD analyses. Untreated sugar cane bagasse (solid black line) compared with sugar cane bagasse pretreated with acidified EC/EG (dotted black line), BMIMCl (black dashed line), and ball-milling (solid grey line). Additional file 5: Figure S5. Glucan conversion in H_2_SO_4_ steam-exploded bagasse by Celluclast 1.5 L at a dosage of 4 FPU/g glucan with the addition of corn stover leaf extracts from transgenic (closed symbols) and non-transgenic (open symbols) events prepared at 16:1 (squares), 12:1 (diamonds), and 8:1 (triangles) buffer-to-dry mass ratios. The numeral 1on the *x*-axis represents the total MULase activity present in Celluclast 1.5 L at a dosage of 4 FPU/g glucan. Values above 1 indicate the addition of corn stover leaf extract containing recombinant CBH to Celluclast 1.5 L in units of MULase activity equal to the total MULase activity in Celluclast 1.5 L at 4 FPU/g glucan. Glucose release from cellulose was monitored using a colorimetric (GOPOD) assay and the results reported as the percentage of glucan converted to glucose. Three samples were analysed per time point; error bars indicate standard deviation. Additional file 6: Figure S6. Cellic CTec2 dose responses on pretreated bagasse at 0.65% (w/v) glucan. (a) H_2_SO_4_ and steam explosion. (b) NaOH and steam explosion. (c) Glycerol/HCl and steam explosion. (d) Acidified EC/EG. (e) BMIMCl. (f) Ball-milling. Three samples were analysed per time point; error bars indicate standard deviation. There was no significant conversion of glucan to glucose in the absence of cellulase. Additional file 7: Figure S7. Corn stover leaf fragments generated by recycling through a Retsch SM 100 cutting mill. (a) Three passes. (b) Six passes. (c) Nine passes.

## Abbreviations

BMIMCl: 1-butyl-3-methylimidazolium chloride
CBH: cellobiohydrolase
FTIR: Fourier transform infrared
SEM: scanning electron microscopy
XRD: X-ray diffraction
