# Supplementary material for: The combination of plant-expressed cellobiohydrolase and low dosages of cellulases for the hydrolysis of sugar cane bagasse
Source: Biotechnol Biofuels. 2014 Sep 9;7:131. doi: 10.1186/s13068-014-0131-9 (PMC4172943; doi:10.1186/s13068-014-0131-9)
Supplement: Additional file 1: Figure S1. — Amino acid sequence of recombinant CBH. The N-terminal signal peptide is underlined. The C-terminal vacuolar sorting determinant is double-underlined. [file 13068_2014_131_MOESM1_ESM.doc]

MRVLLVALALLALAASATSQQIGTYTAETHPSLSWSTCKSGGSCTTNSGAITLDANWRWVHGVNTSTNCYTGNTWNTAICDTDASCAQDCALDGADYSGTYGITTSGNSLRLNFVTGSNVGSRTYLMADNTHYQIFDLLNQEFTFTVDVSHLPCGLNGALYFVTMDADGGVSKYPNNKAGAQYGVGYCDSQCPRDLKFIAGQANVEGWTPSSNNANTGLGNHGACCAELDIWEANSISEALTPHPCDTPGLSVCTTDACGGTYSSDRYAGTCDPDGCDFNPYRLGVTDFYGSGKTVDTTKPITVVTQFVTDDGTSTGTLSEIRRYYVQNGVVIPQPSSKISGVSGNVINSDFCDAEISTFGETASFSKHGGLAKMGAGMEAGMVLVMSLWDDYSVNMLWLDSTYPTNATGTPGAARGSCPTTSGDPKTVESQSGSSYVTFSDIRVGPFNSTFSGGSSTGGSSTTTASGTTTTKASSTSTSSTSTGTGVAAHWGQCGGQGWTGPTTCASGTTCTVVNPYYSQCLDELKAEAK
