# Supplementary figures and images for: The combination of plant-expressed cellobiohydrolase and low dosages of cellulases for the hydrolysis of sugar cane bagasse
Source: Biotechnol Biofuels. 2014 Sep 9;7:131. doi: 10.1186/s13068-014-0131-9 (PMC4172943; doi:10.1186/s13068-014-0131-9)

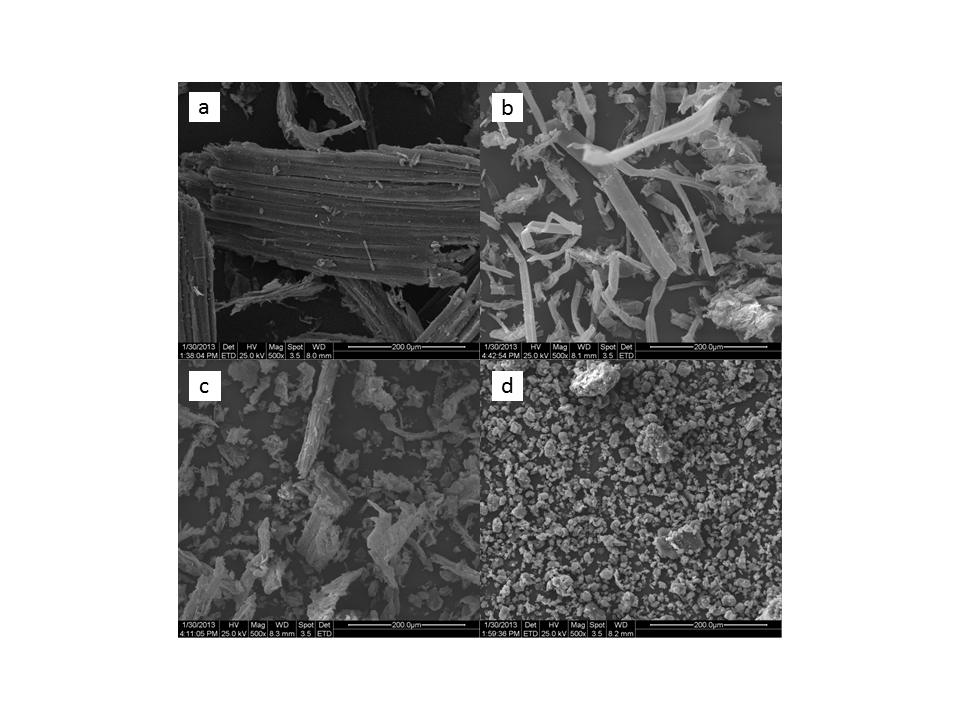

Supplement: Additional file 2: Figure S2. — SEM analysis. (a) Untreated sugar cane bagasse and sugar cane bagasse pretreated with (b) acidified EC/EG, (c) BMIMCl, and (d) ball-milling. [file 13068_2014_131_MOESM2_ESM.png]

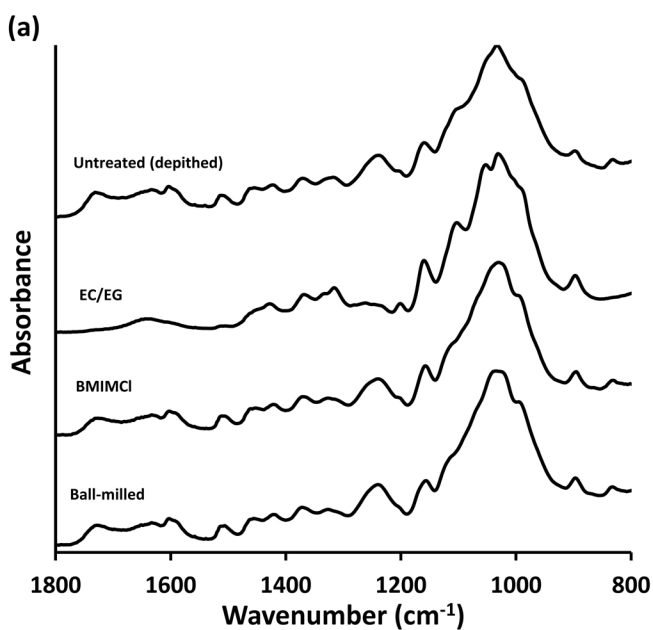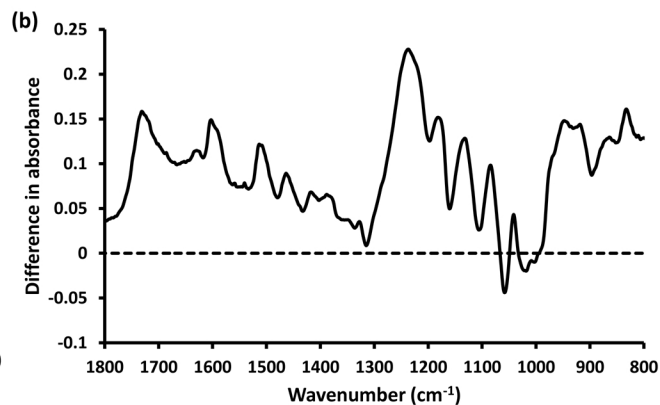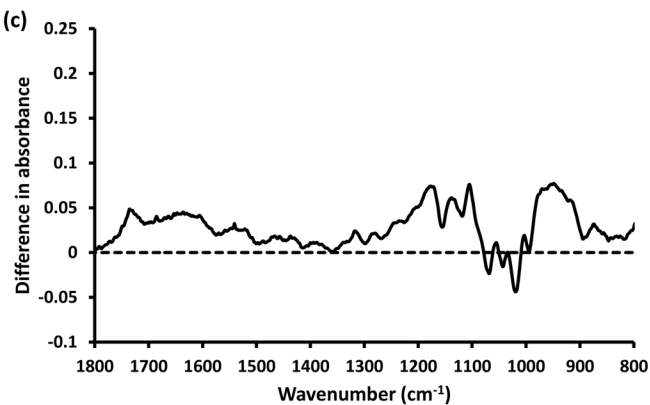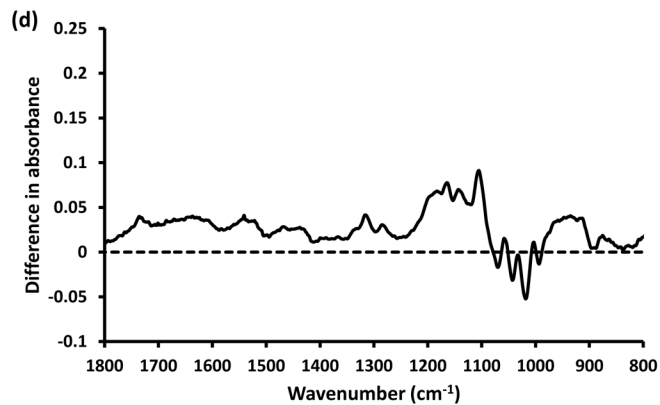

Supplement: Additional file 3: Figure S3. — FTIR analysis. (a) FTIR spectra of untreated sugar cane bagasse and comparison with sugar cane bagasse pretreated with acidified EC/EG, BMIMCl, and ball-milling. FTIR difference spectra between untreated (depithed) bagasse and bagasse pretreated with (b) acidified EC/EG, (c) BMIMCl, and (d) ball-milling. A positive value in the FTIR difference spectra corresponds to the loss of a spectral feature relative to untreated bagasse. [file 13068_2014_131_MOESM3_ESM.pdf]

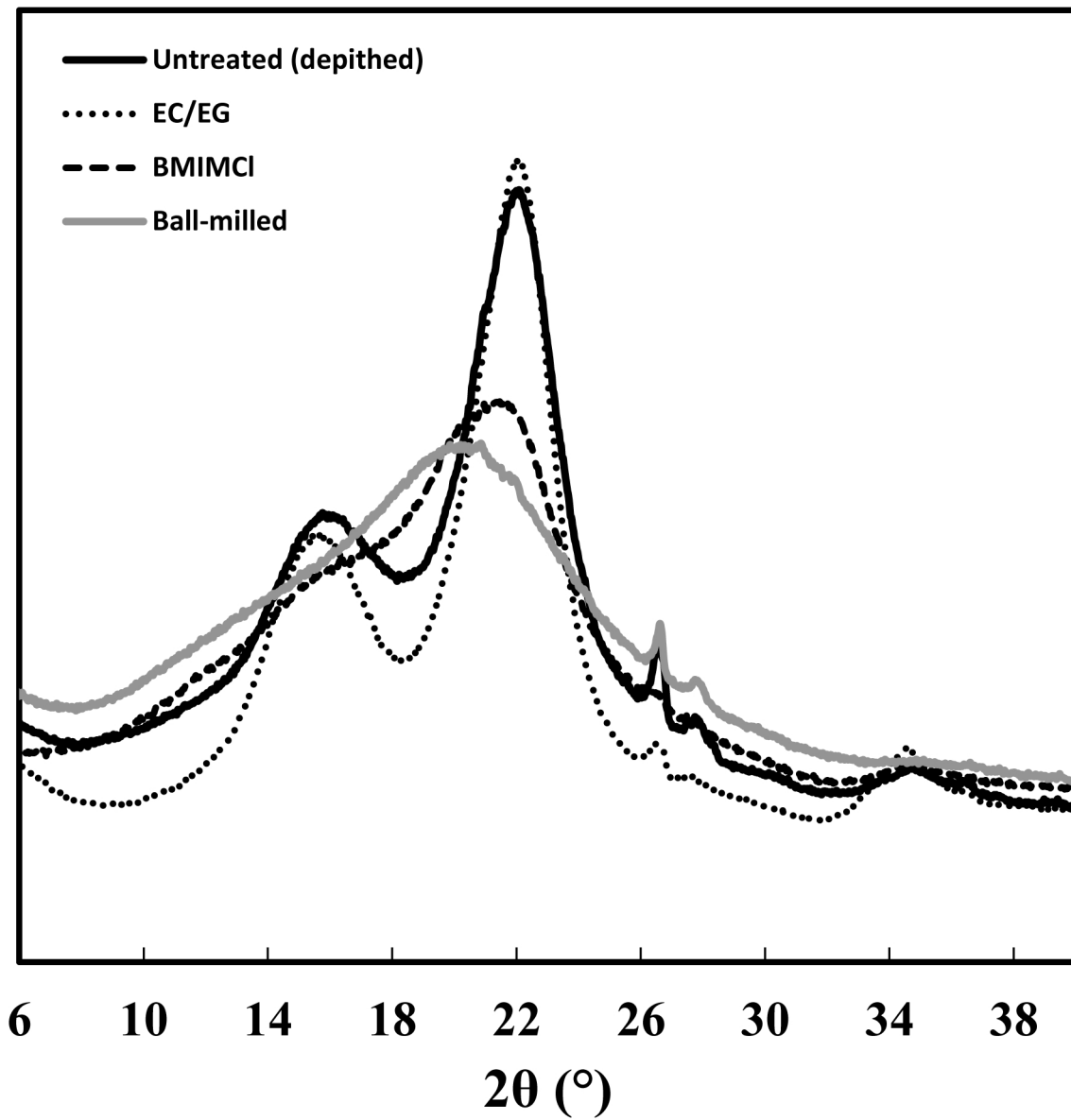

Supplement: Additional file 4: Figure S4. — XRD analyses. Untreated sugar cane bagasse (solid black line) compared with sugar cane bagasse pretreated with acidified EC/EG (dotted black line), BMIMCl (black dashed line), and ball-milling (solid grey line). [file 13068_2014_131_MOESM4_ESM.pdf]

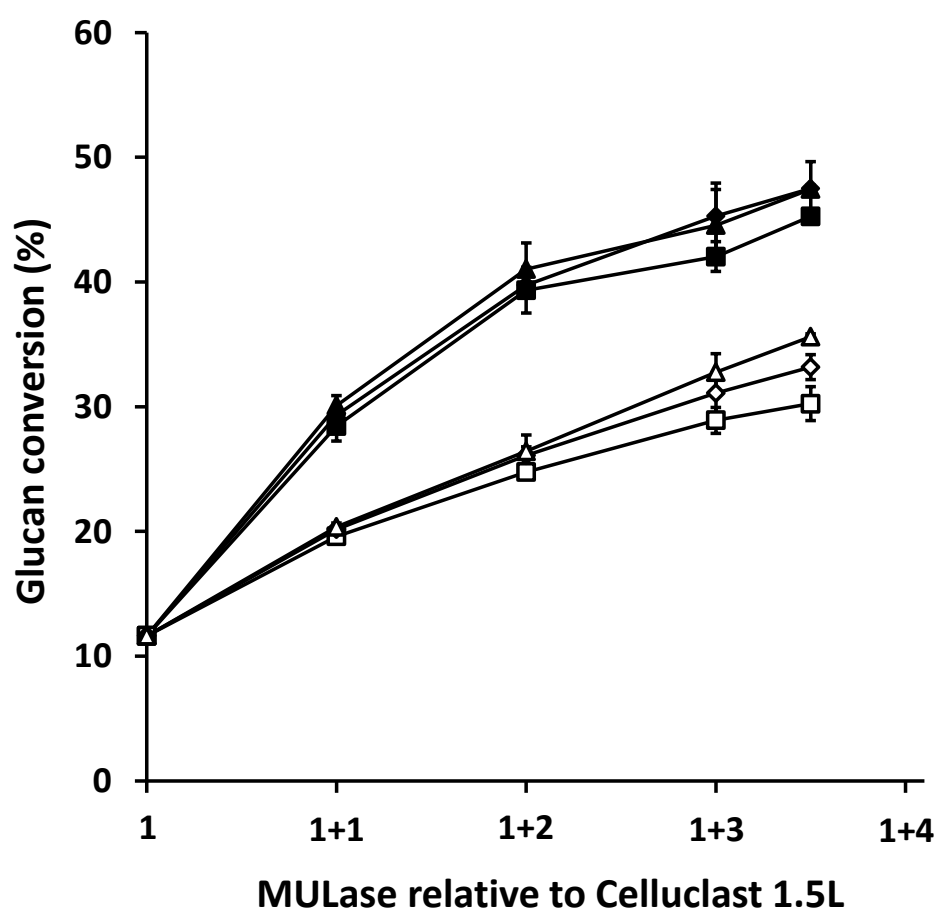

Supplement: Additional file 5: Figure S5. — Glucan conversion in H2SO4 steam-exploded bagasse by Celluclast 1.5 L at a dosage of 4 FPU/g glucan with the addition of corn stover leaf extracts from transgenic (closed symbols) and non-transgenic (open symbols) events prepared at 16:1 (squares), 12:1 (diamonds), and 8:1 (triangles) buffer-to-dry mass ratios. The numeral 1on the x-axis represents the total MULase activity present in Celluclast 1.5 L at a dosage of 4 FPU/g glucan. Values above 1 indicate the addition of corn stover leaf extract containing recombinant CBH to Celluclast 1.5 L in units of MULase activity equal to the total MULase activity in Celluclast 1.5 L at 4 FPU/g glucan. Glucose release from cellulose was monitored using a colorimetric (GOPOD) assay and the results reported as the percentage of glucan converted to glucose. Three samples were analysed per time point; error bars indicate standard deviation. [file 13068_2014_131_MOESM5_ESM.pdf]

**(a)**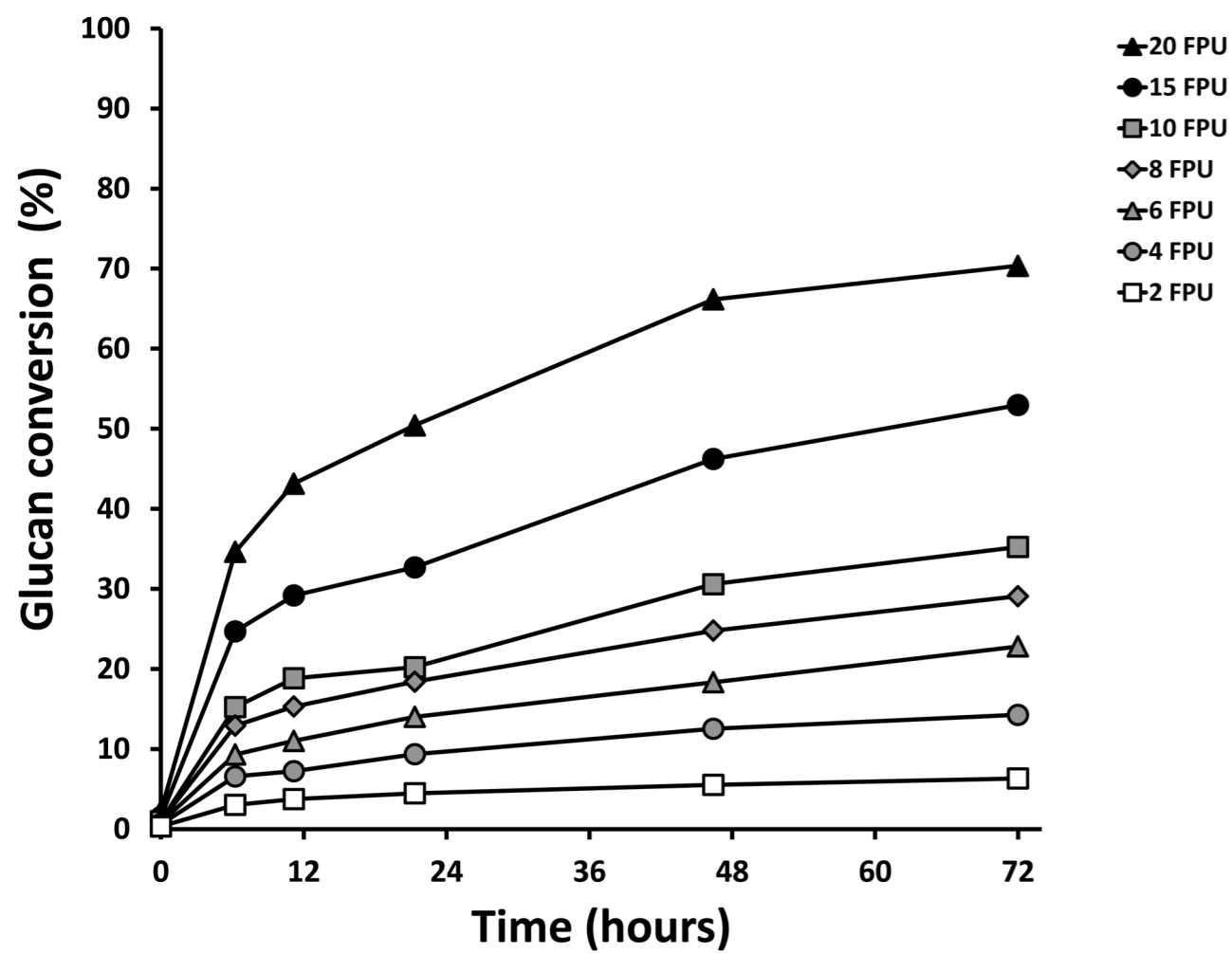**(b)**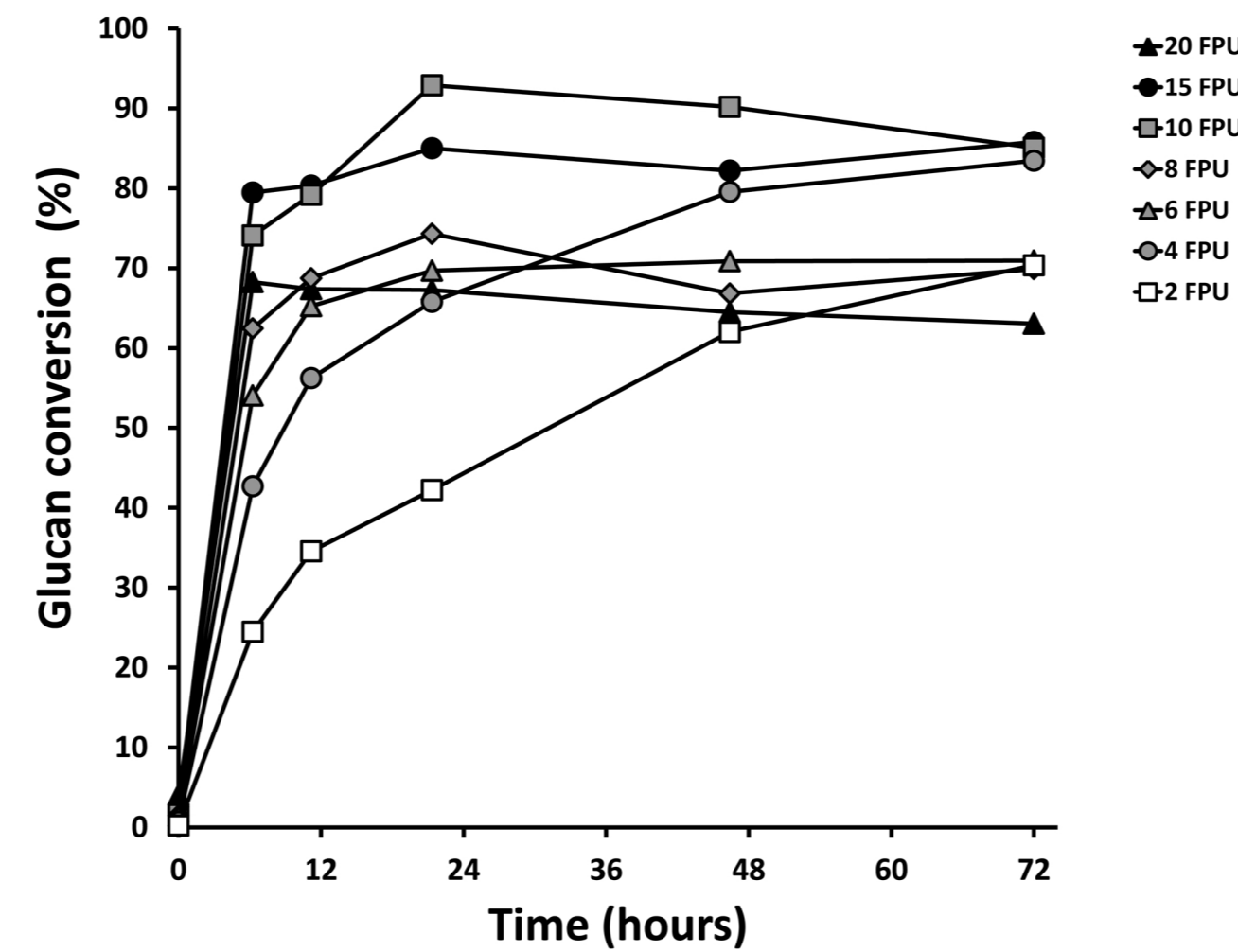**(c)**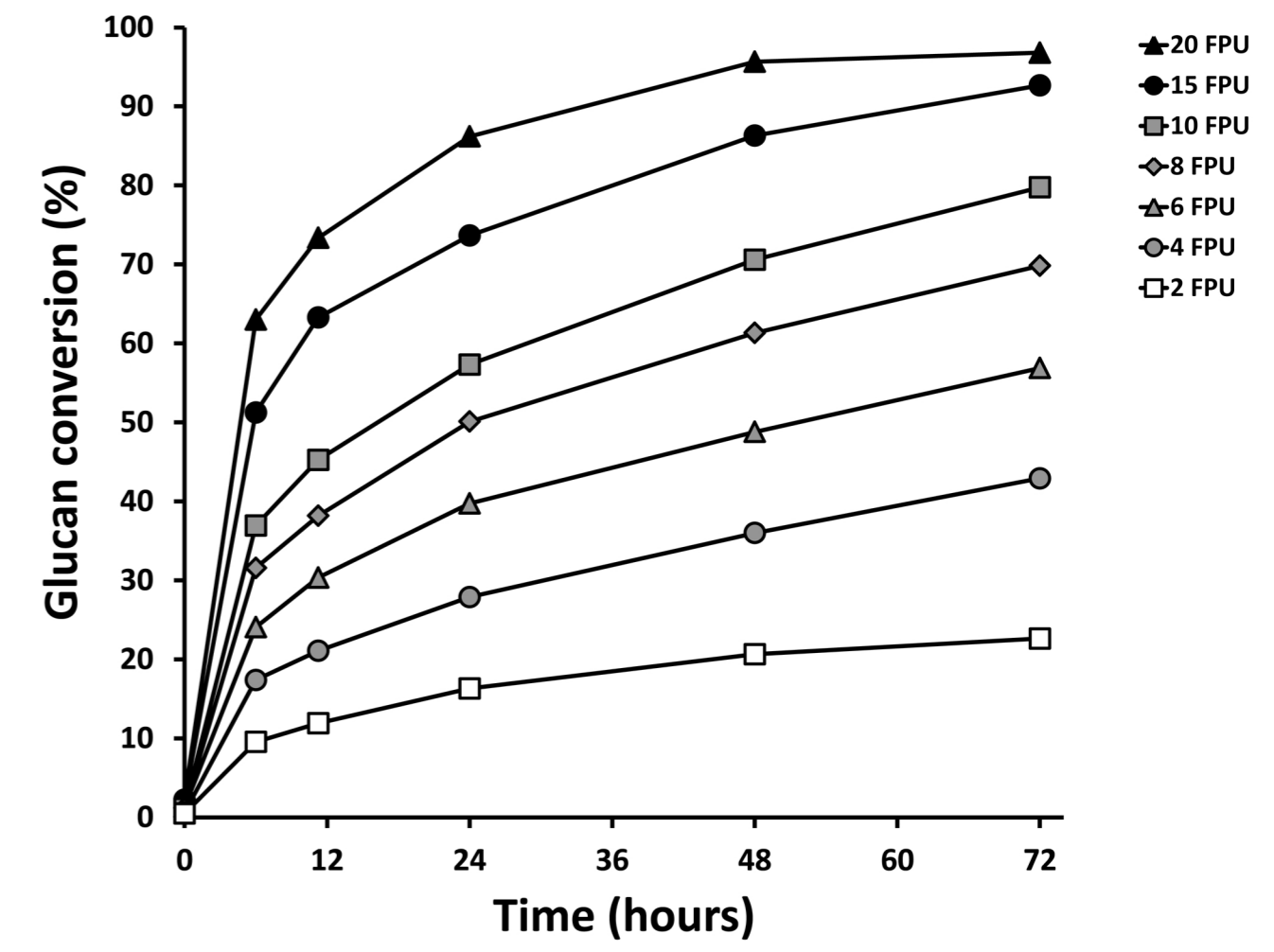**(d)**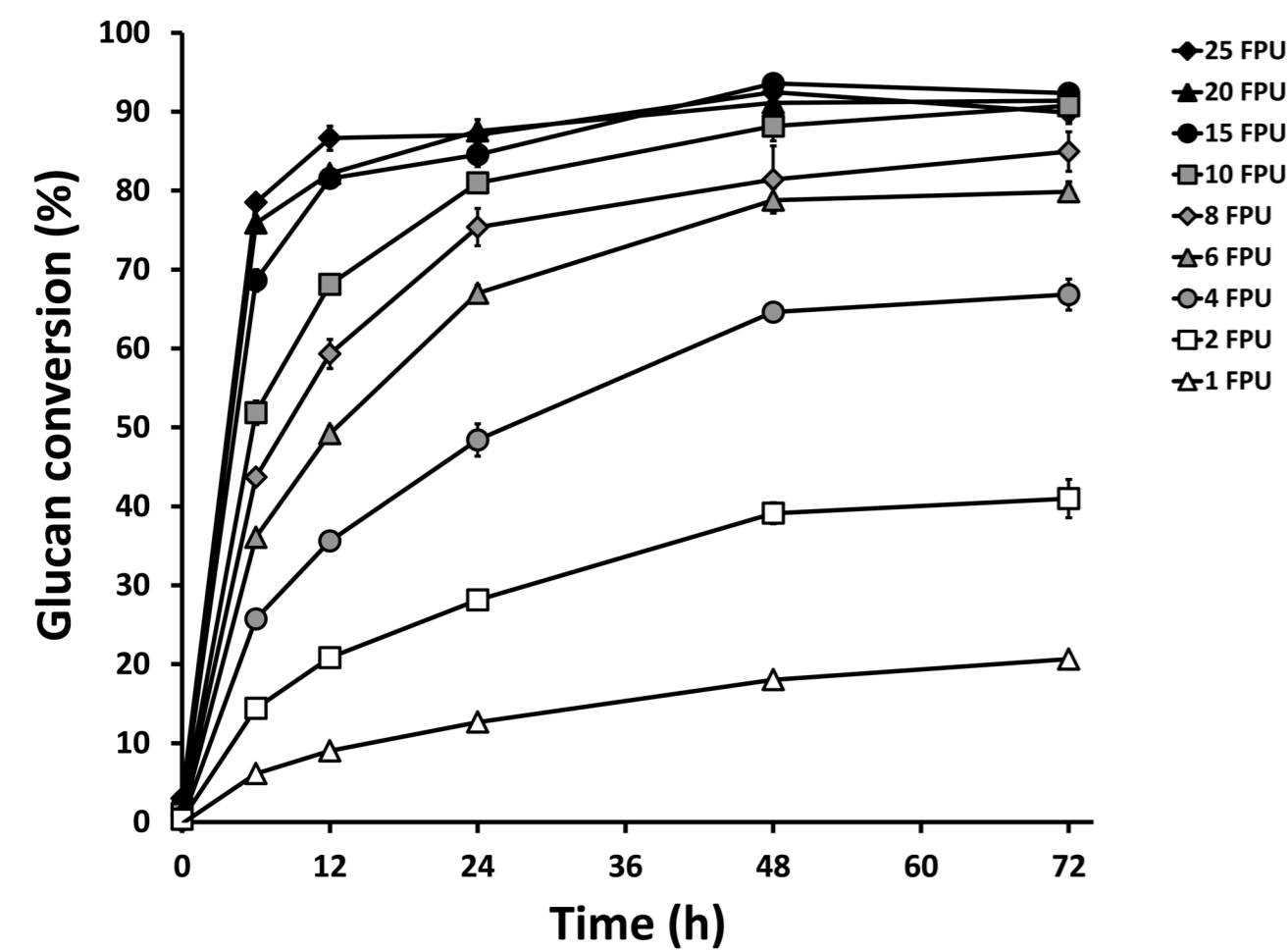**(e)**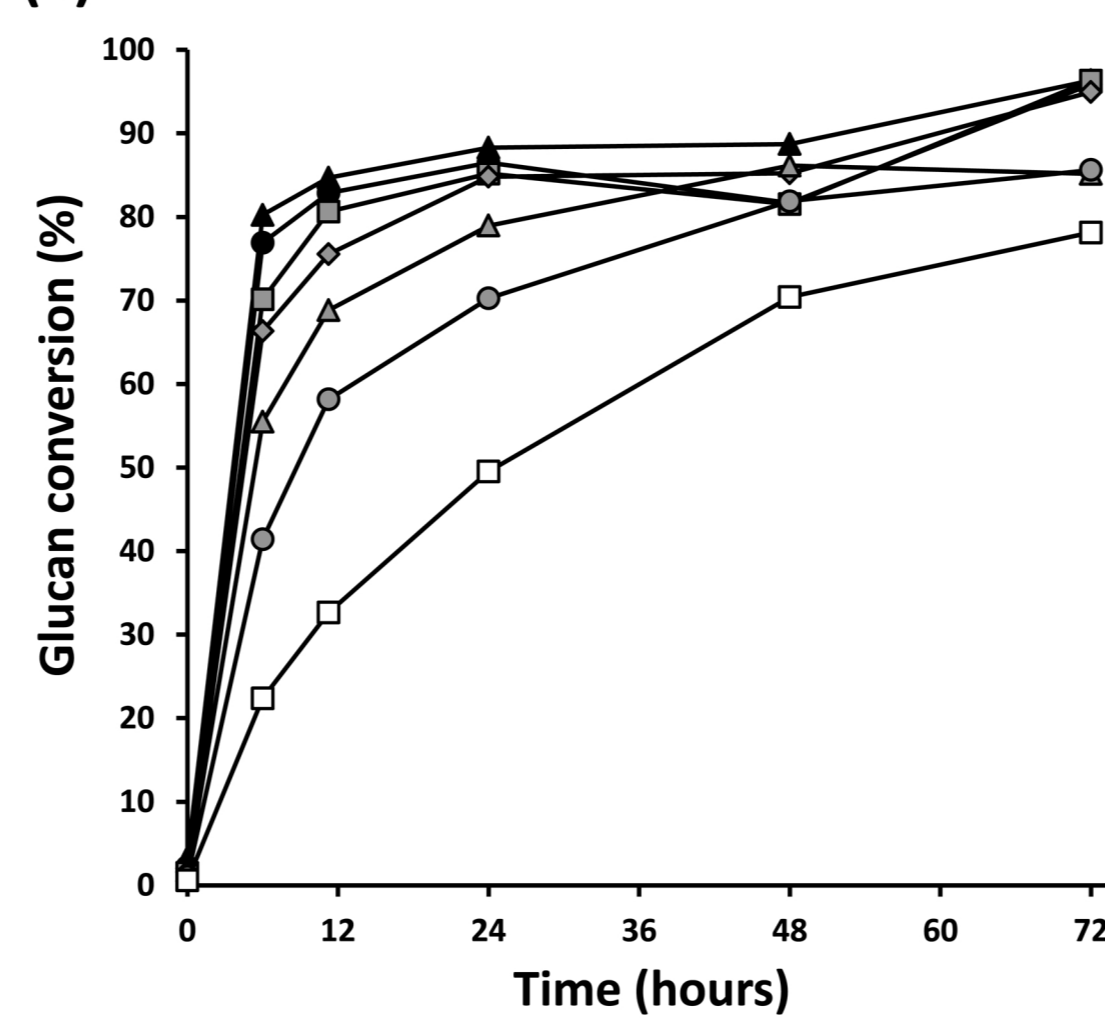**(f)**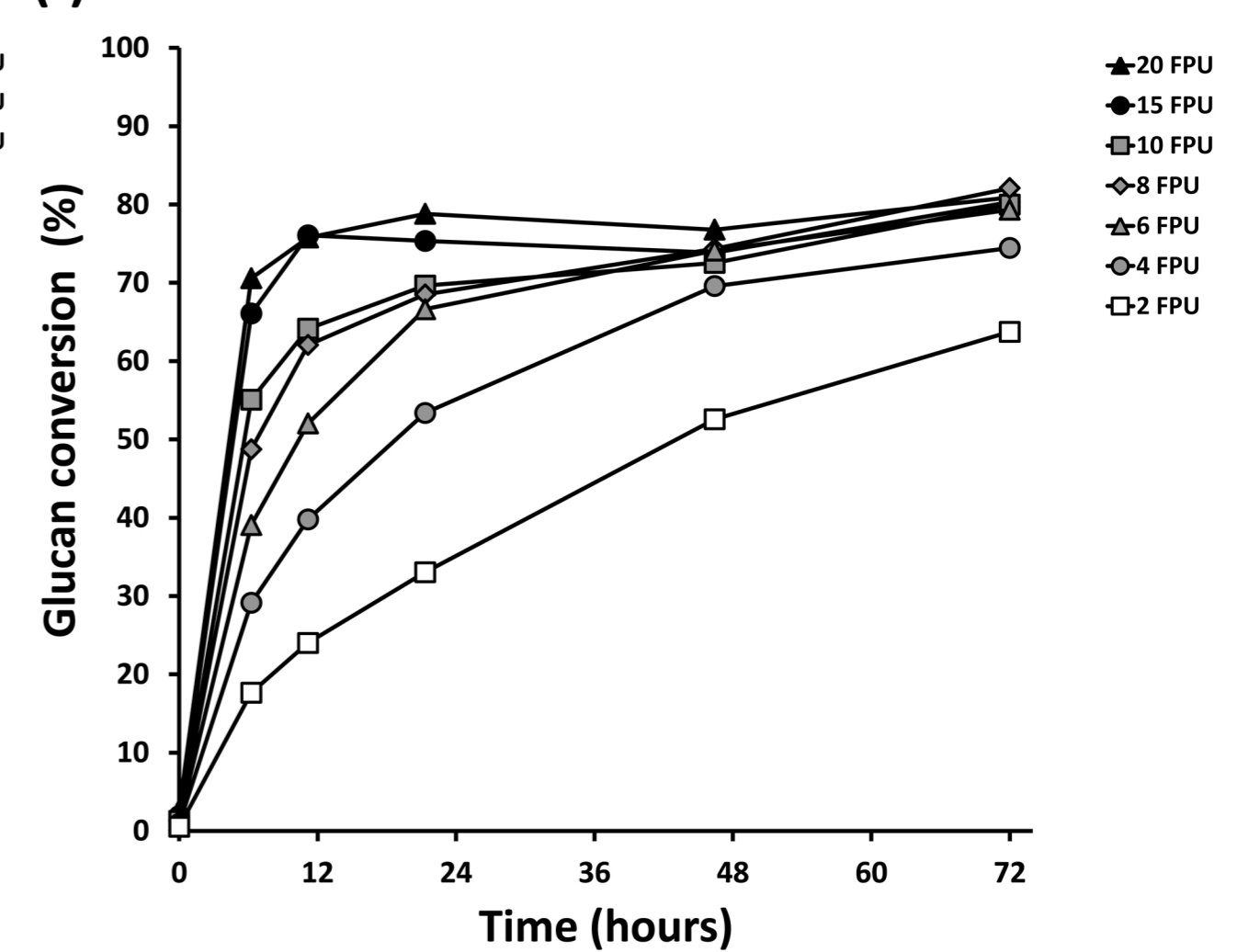

Supplement: Additional file 6: Figure S6. — Cellic CTec2 dose responses on pretreated bagasse at 0.65% (w/v) glucan. (a) H2SO4 and steam explosion. (b) NaOH and steam explosion. (c) Glycerol/HCl and steam explosion. (d) Acidified EC/EG. (e) BMIMCl. (f) Ball-milling. Three samples were analysed per time point; error bars indicate standard deviation. There was no significant conversion of glucan to glucose in the absence of cellulase. [file 13068_2014_131_MOESM6_ESM.pdf]

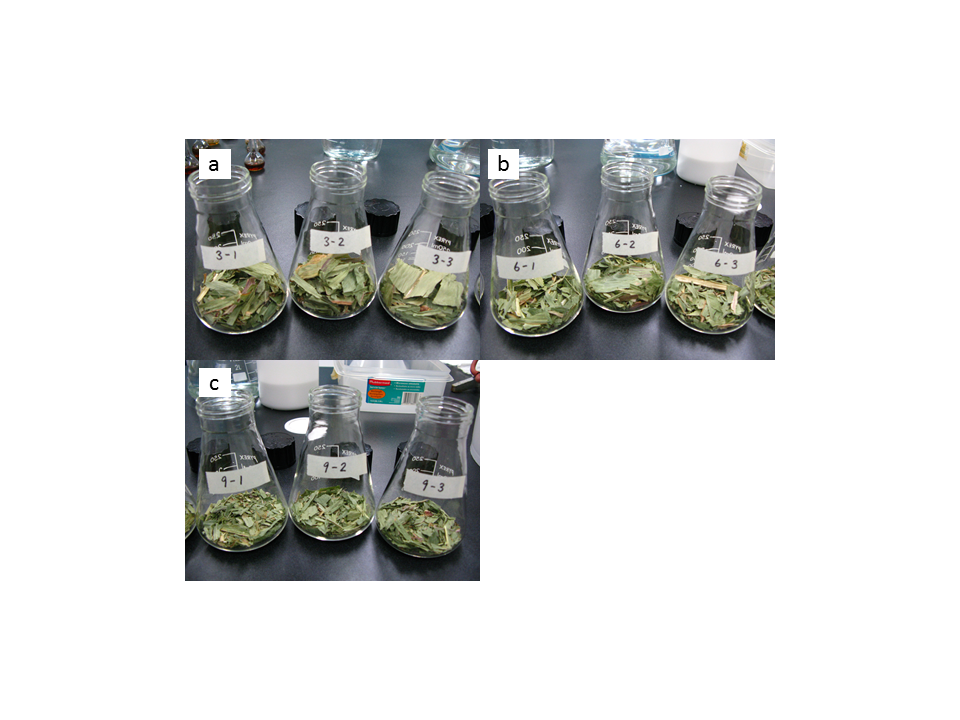

Supplement: Additional file 7: Figure S7. — Corn stover leaf fragments generated by recycling through a Retsch SM 100 cutting mill. (a) Three passes. (b) Six passes. (c) Nine passes. [file 13068_2014_131_MOESM7_ESM.png]
